# Supplementary material for: Co-design to consensus: Identifying the core elements of a novel intervention for pre-school children with co-occurring phonological speech sound disorder (SSD) and developmental language disorder (DLD) using a modified e-Delphi approach
Source: PLoS One. 2025 Jun 18;20(6):e0326072. doi: 10.1371/journal.pone.0326072 (PMC12176183; doi:10.1371/journal.pone.0326072)
Supplement: S2 — (DOCX) [file pone.0326072.s002.docx]

Adapted GRIPP 2 Short Form

| **Section and item** | **Where stated within the article** | **Further elaboration/additional information** |
| --- | --- | --- |
| **Aim:** Aim of PPI in the study | “The current study” section of the introduction. | N/A |
| **Methods:** Clear description of the methods used for PPI in the study | Part 1 of the methods section (co-design of core elements).  ‘Review process and rewording’ section of the results. | N/A |
| **Study results:** Outcomes- reported results of PPI in the study, including positive and negative outcomes | ‘Review process and rewording’ section of the results.  Table 3 of re-worded statements.  Paragraph on final rates of consensus reached at the end of the results. | N/A |
| **Discussion and conclusions:** Outcomes—Comment on the extent to which PPI influenced the study overall. Describe positive and negative effects | Strengths and limitations section of the discussion. | N/A |
| **Reflections/critical perspective:** Comment critically on the study, reflecting on the things that went well and those that did not, so others can learn from this experience | Strengths and limitations section of the discussion. | A separate commentary article is currently being written up by steering group members. The article goes into detail about their personal reflections of being involved in the intervention development process (across all 4 study phases). |
