# Supplementary material for: Co-design to consensus: Identifying the core elements of a novel intervention for pre-school children with co-occurring phonological speech sound disorder (SSD) and developmental language disorder (DLD) using a modified e-Delphi approach
Source: PLoS One. 2025 Jun 18;20(6):e0326072. doi: 10.1371/journal.pone.0326072 (PMC12176183; doi:10.1371/journal.pone.0326072)
Supplement: S3 — (DOCX) [file pone.0326072.s003.docx]

**S3: ‘Pre-design’ steering group activities**

***1.Interactive whiteboard- systematic review findings discussion***


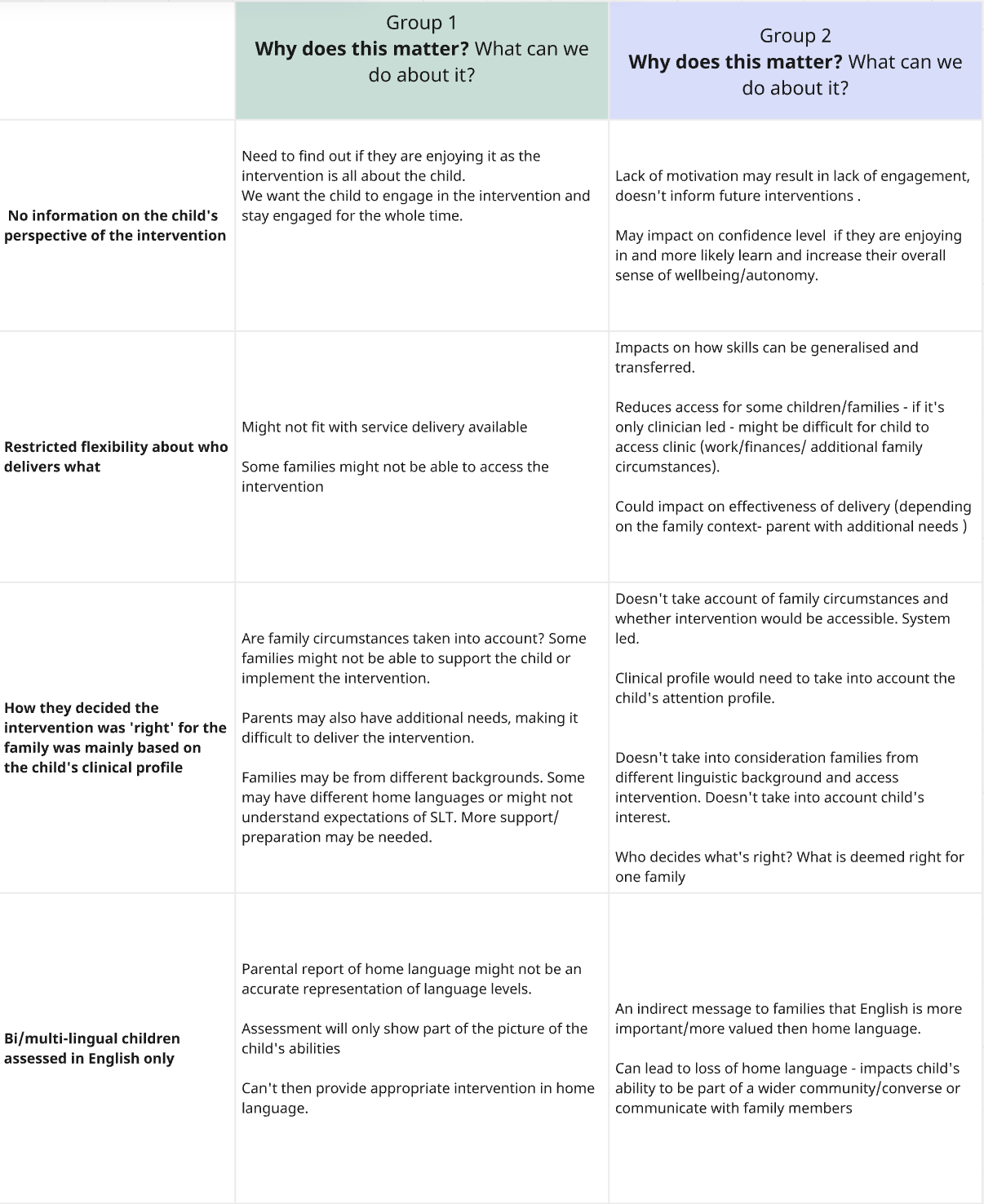


***2. Survey findings document - used as a ‘probe’ for 1:1 discussion***

We asked speech and language therapists (SLTs) **how they would provide an intervention** for **both** language (vocabulary) and speech, in young children.

There were **119 responses**.

We had responses from SLTs across the **UK and Northern Ireland**.

Most worked for the **NHS**. Some also worked in education settings and privately.

In blue is a comparison with the systematic review findings.

**Content**

**1.** Survey: For language, many clinicians would prioritise targeting **language of importance** to the **child/everyday life**.

Systematic review: some evidence for this, mostly with parent-child interaction based interventions.

**2.**Survey: For speech, many clinicians would target sounds which have the **most impact** on the child being **understood** by others.

Systematic review: speech interventions tended to focus on specific processes/norms rather than impact on intelligibility.

**3.**Survey: For both speech and language, SLTs did **not** prioritise targets based on **‘developmental norms’**.

Systematic review: For both speech and language interventions, most targets were based on developmental norms.

**4.**Survey: For language, the technique of **labelling (including modelling)** was a clear preference.

Systematic review: Modelling was the top technique mentioned in language interventions.

**5.**Survey: There were no clear preferences for speech techniques. Techniques relating to **sound exposure** were ranked most highly though.

Systematic review: Some interventions looked at sound exposure through recasting, which came out as a top speech technique.

**6.**Survey: All SLTs said they would use general strategies to support others in **understanding what the child is saying (intelligibility)** in everyday life. This includes asking the child to **‘show them’**, and **picture boards**.

Systematic review: General strategies to improve intelligibility in everyday life not mentioned within the speech interventions.

**7.**Survey: Many SLTs said that they would also use techniques to **elicit speech output,** but this was very dependent on the child’s ‘**readiness’**.

Systematic review: ‘readiness’ in terms of the child’s attention and confidence not mentioned within the speech studies.

**8.**Survey: Significant **overlaps** between **activities** for speech and language.

Systematic review: Found similar overlaps.

**9.**Survey: Most SLTs would also work on ‘**phonological awareness’**. About half would do **general phonological awareness**, with the other half doing phonological awareness work **specific to the sounds** the child struggles with.

Systematic review: N/A

**Format**

**10.**Survey: Most SLTs preferred to work on speech and language targets at the **same time** (e.g. in the same session).

Systematic review: N/A

**Delivery**

**11.**Survey: Most SLTs preferred a **range of people** to deliver the intervention techniques, not just the SLT.

Systematic review: Speech and language interventions tended to be professional led or through a trained parent. Some hybrid parent/professional working, mainly in the language interventions.

**12.**Survey: Most SLTs preferred for the intervention techniques to be used in a **range of settings**, not just in a clinic.

Systematic review: Language interventions placed more emphasis on technique use at home. Speech interventions tended to focus on technique use in clinic.

**13.**Survey: Many clinicians would like to know what **‘dosage’** the techniques are provided in. For example, **how many times** a technique should be used per therapy activity.

Systematic review: Dosage often under-specified.

**14.**Survey: All SLTs said they would **support significant others** in using an intervention technique by **modelling it** to them. Providing **written guidance**, and identifying the **best time** to use these techniques in everyday life, were also popular.

Systematic review: These strategies are also mentioned in the two speech and language interventions which involved a trained parent.
