# Supplementary material for: Co-design to consensus: Identifying the core elements of a novel intervention for pre-school children with co-occurring phonological speech sound disorder (SSD) and developmental language disorder (DLD) using a modified e-Delphi approach
Source: PLoS One. 2025 Jun 18;20(6):e0326072. doi: 10.1371/journal.pone.0326072 (PMC12176183; doi:10.1371/journal.pone.0326072)

**S4:‘Generative’ co-design activities**

***Miro boards x 2- potential intervention characteristics and steering group ideas***


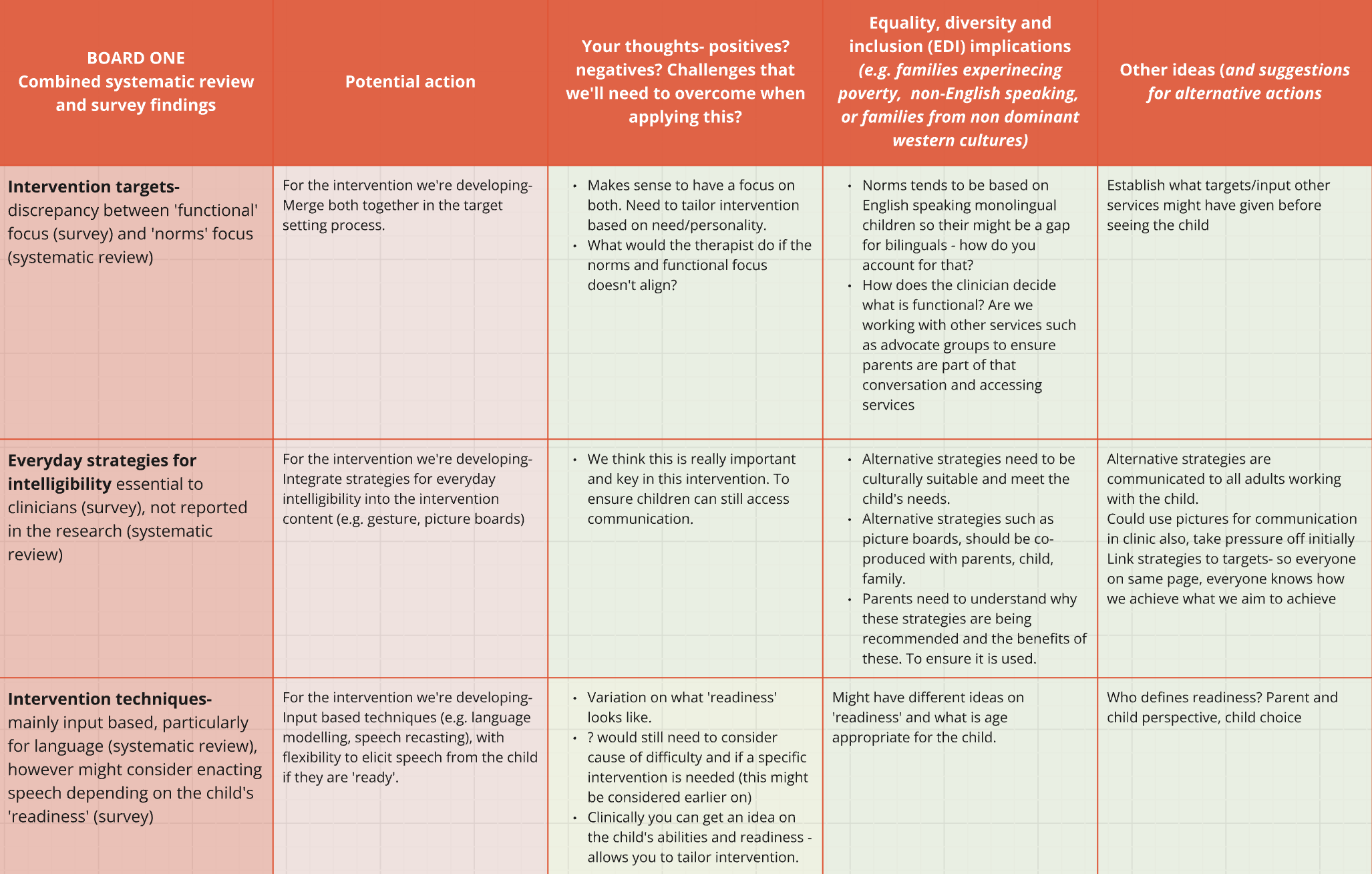


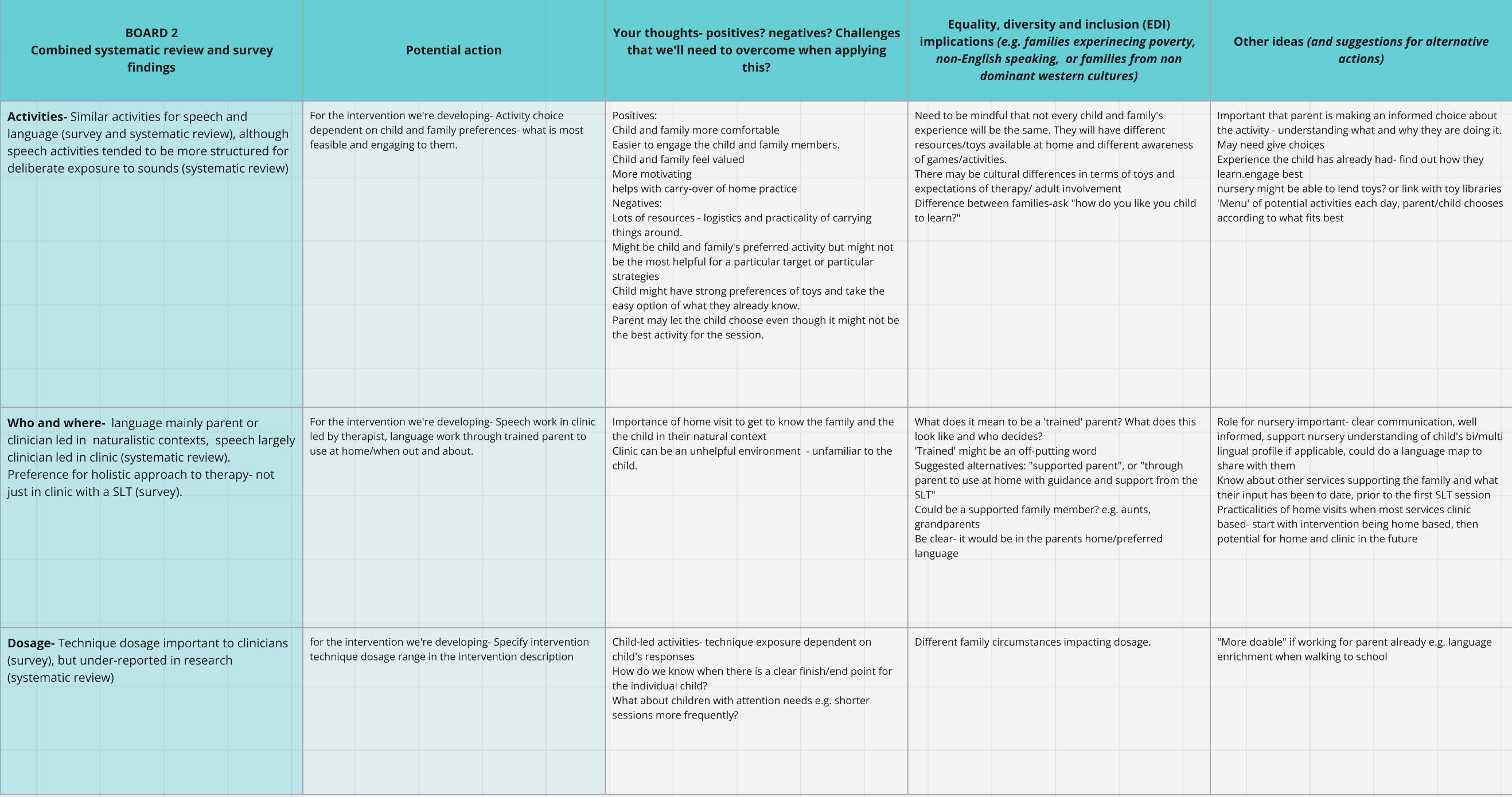

Supplement: S4 — (DOCX) [file pone.0326072.s004.docx]
