# Supplementary material for: Co-design to consensus: Identifying the core elements of a novel intervention for pre-school children with co-occurring phonological speech sound disorder (SSD) and developmental language disorder (DLD) using a modified e-Delphi approach
Source: PLoS One. 2025 Jun 18;20(6):e0326072. doi: 10.1371/journal.pone.0326072 (PMC12176183; doi:10.1371/journal.pone.0326072)
Supplement: S5 — (DOCX) [file pone.0326072.s005.docx]

**S5: Behaviour change techniques mapped onto the behaviour change technique ontology**

***Table 1. Behaviour change techniques approved by the project steering group***

| **BCT from BCTv1** (Barnett et al., 2023) | **Definition on BCTv1** | **Corresponding BCTO item** |
| --- | --- | --- |
| 1.1 Goal-setting (behaviour) | Set or agree on a goal defined in terms of the behaviour to be achieved | **Set behaviour goal BCT**  <https://bciosearch.org/BCIO_007003>  A goal setting BCT that sets a goal for the behaviour to be achieved. |
| 1.2 Problem-solving | Analyse, or prompt the person to analyse, factors influencing the behaviour and generate or select strategies that include overcoming barriers and/or increasing facilitators (includes ‘Relapse Prevention’ and ‘Coping Planning’) | **Goal strategizing BCT**  <https://bciosearch.org/BCIO_007008>  A goal directed BCT in which the person analyses factors influencing the behaviour and generates, selects, or reviews strategies to increase facilitators and overcome barriers |
| 1.3 Goal-setting (outcome) | Set or agree on a goal defined in terms of a positive outcome of wanted behaviour | **Agree outcome goal BCT**  <https://bciosearch.org/BCIO_007006>  A goal setting BCT that involves the intervention source agreeing with the person on a goal which is a positive outcome of performing the behaviour. |
| 1.4 Action-planning | Prompt detailed planning of performance of the behaviour (must include at least one of context, frequency, duration and intensity). Context may be environmental (physical or social) or internal (physical, emotional or cognitive) (includes ‘Implementation Intentions’) | **Action planning BCT**  <https://bciosearch.org/BCIO_007010>  A goal setting BCT that involves the intervention source agreeing with the person on a goal which is a positive outcome of performing the behaviour. |
| 2.2. Feedback on behaviour | Monitor and provide informative or evaluative feedback on performance of the behaviour (e.g., form, frequency, duration, intensity) | **Provide feedback BCT**  <https://bciosearch.org/BCIO_007022>  A monitoring BCT in which feedback about the behaviour is provided. |
| 2.3 Self-monitoring of behaviour | Establish a method for the person to monitor and record their behaviour(s) as part of a behaviour change strategy | **Self monitor behaviour BCT**  <https://bciosearch.org/BCIO_007024>  A monitoring BCT in which the person uses a method to monitor and record their behaviour. |
| 2.4. Self-monitoring of outcome(s) of behaviour | Establish a method for the person to monitor and record the outcome(s) of their behaviour as part of a behaviour change strategy | **Self-monitor outcome of behaviour BCT**  <https://bciosearch.org/BCIO_007025>  A monitoring BCT in which the person uses a method to monitor and record an outcome of their behaviour. |
| 2.7. Feedback on outcome(s) of behaviour | Monitor and provide feedback on the outcome of performance of the behaviour | **Provide feedback on outcome of behaviour BCT**  <https://bciosearch.org/BCIO_007027>  A provide feedback BCT that provides information about an outcome of the person's previous performance of the behaviour. |
| 3.1 Social support (unspecified) | Advise on, arrange or provide social support (e.g., from friends, relatives, colleagues, ’buddies’ or staff) or non-contingent praise or reward for performance of the behaviour. It includes encouragement and counselling, but only when it is directed at the behaviour | **Social Support BCT**  <https://bciosearch.org/BCIO_007028>  A behaviour change technique that involves taking steps to secure or deliver the support or aid of another person. |
| 5.3 Information about social and environmental consequences | Provide information (e.g., written, verbal, visual) about social and environmental consequences of performing the behaviour | **Inform about social consequences BCT**  <https://bciosearch.org/BCIO_007064>  An increased awareness of consequences BCT that provides information about the social consequences of performing or not performing the behaviour.  **Inform about environmental consequences BCT**  <https://bciosearch.org/BCIO_007176>  An increased awareness of consequences BCT that provides information about the environmental consequences of performing or not performing the behaviour. |
| 6.1 Demonstration of target behaviour | Provide an observable sample of the performance of the behaviour, directly in person or indirectly e.g., via film, pictures, for the person to aspire to or imitate (includes ‘Modelling’) | **Demonstrate the behaviour BCT**  <https://bciosearch.org/BCIO_007055>  A guide how to perform behaviour BCT that provides an observable sample of the performance of the behaviour for the person to aspire to or imitate. |
| 7.3 Reduce prompts/cues | Withdraw gradually prompts to perform the behaviour (includes ‘Fading’) | Child class under ‘associative learning BCT’  **Reduce cue frequency BCTs**  <https://bciosearch.org/BCIO_007084>  An alter external stimulus BCT in which cues for the behaviour are presented less frequently. |
| 8.1 Behavioural practice and rehearsal | Prompt practice or rehearsal of the performance of the behaviour one or more times in a context or at a time when the performance may not be necessary, in order to increase habit and skill | **Skill development behaviour**  <https://bciosearch.org/BCIO_036039>  A learning behaviour that involves improving the ability to perform physical, psychological or social activities.  **Knowledge development behaviour**  <https://bciosearch.org/BCIO_050291>  A learning behaviour that involves improving a person's knowledge. |
| 8.6 Generalization of target behaviour | Advise to perform the wanted behaviour, which is already performed in a particular situation, in another situation | **Generalisation in learning**  <https://bciosearch.org/BCIO_006124>  Associative learning process in which prior learning that an antecedent stimulus signals certain consequences of behaviour spreads to a stimulus that differs in certain aspects from the original antecedent stimulus. |
| 10.4 Social reward (behaviour) | Arrange a verbal or non-verbal reward if and only if there has been effort and/or progress in performing the behaviour (includes ‘Positive reinforcement’) | **Provide positive social consequence for the behaviour BCT**  <https://bciosearch.org/BCIO_007265>  A provide positive consequence for behaviour BCT where the consequence is an interpersonal process or a proxy interpersonal process. |
| 12.5 Adding objects to the environment | Add objects to the environment in order to facilitate performance of the behaviour | **Add objects to the environment BCT**  <https://bciosearch.org/BCIO_007156>  A restructure the physical environment BCT that adds objects to the person's physical surroundings. |

***Table 2. Behaviour change techniques excluded by the steering group***

| **BCT from BCTv1** (Barnett et al., 2023) | **Definition on BCTv1** | **Corresponding BCTIO item** | **Rationale** |
| --- | --- | --- | --- |
| 2.1. Monitoring of behaviour by others without feedback | Observe or record behaviour with the person's knowledge as part of a behaviour change strategy | **Record behaviour without feedback BCT**  <http://humanbehaviourchange.org/ontology/BCIO_007019>  A monitoring BCT that records current performance of the behaviour with the person’s knowledge but without providing feedback about their behaviour. | Best to share this feedback with the parent so they can learn from it.  Example quotes from impact log:  “Pointless”  “Makes no sense, it’s like putting them under exam” |
| 2.5. Monitoring of outcome(s) of behaviour without feedback | Observe or record outcomes of behaviour with the person's knowledge as part of a behaviour change strategy | **Observe outcome of behaviour without feedback BCT**  <https://bciosearch.org/BCIO_007020>  A monitoring BCT that observes an outcome of performing the behaviour with the person’s knowledge but without providing feedback about the outcome. | Parent would benefit from knowing what the clinician has observed. Not much to gain from the clinician noting this but not sharing it.  Example quote from impact log:  “it’s irrelevant” |
| 4.1 Instruction on how to perform behaviour | Advise or agree on how to perform the behaviour (includes ‘Skills training’) | **Instruct how to perform a behaviour BCT**  <https://bciosearch.org/BCIO_007058>  A guide how to perform behaviour BCT that involves telling the person how to perform the behaviour. | Very similar to “action planning” BCT which is worded better- use that instead.  Example quote from impact log:  “it’s one way” |
| 6.3 Information about others’ approval | Provide information about what other people think about the behaviour. The information clarifies whether others will like, approve or disapprove of what the person is doing or will do | **Increase awareness of others’ approval BCT**  <https://bciosearch.org/BCIO_007074>  An awareness of other people's thoughts, feelings and actions BCT that increases awareness of whether others will like, approve, dislike, or disapprove of the behaviour. | Ethicality- could put struggling parents under more pressure. There are other ways that parents could be motivated to carry out the intervention.  Example quote from impact log:  “who do they need approval from?”  “reinforces an unhealthy mindset” |
| 10.2 Material reward (behaviour) | Arrange for the delivery of money, vouchers or other valued objects if and only if there has been effort and/or progress made in performing the behaviour. Note: if the reward is unspecified, code for a non-specific reward | **Provide positive material consequence for behaviour BCT**  <https://bciosearch.org/BCIO_007257>  A provide positive consequence for behaviour BCT where the consequence is money, vouchers or other valued objects. | Unhelpful to have a clinician responsible for deciding whether parents are “deserving”.  Example quotes from impact log:  “that’s not partnership really, it’s power dynamics”  “that’s horrific” |
