# Supplementary material for: Co-design to consensus: Identifying the core elements of a novel intervention for pre-school children with co-occurring phonological speech sound disorder (SSD) and developmental language disorder (DLD) using a modified e-Delphi approach
Source: PLoS One. 2025 Jun 18;20(6):e0326072. doi: 10.1371/journal.pone.0326072 (PMC12176183; doi:10.1371/journal.pone.0326072)
Supplement: S6 — (DOCX) [file pone.0326072.s006.docx]

**S6: Round 1 questionnaire (47 statements)**

E-delphi round 1 (developing a novel pre-school DLD/SSD intervention)

Start of Block: Default Question Block

Thank you again for registering to take part in this e-delphi (2 x approx. 1 hour online surveys).

Reminder: The purpose of the e-delphi is to agree the core elements of a new intervention for pre-school children with co-occurring SSD/DLD features (outcomes: speech comprehensibility/intelligibility and expressive vocabulary).

The strength in this intervention development work is that clinicians like you are playing an active role in what it will look like. 

To recognise your contribution, you will be named in the acknowledgements section of any resulting journal article (if you opted into this during registration). However, we will remind you that your responses will be DE-IDENTIFIED, and you will be de-identified amongst the other survey respondents. 

If you wish to refresh your knowledge of the latest advancements in SSD and DLD, please access the following resources:

 RCSLT SSD clinical guidance
 CATALISE paper

 This is the first survey form for you to complete, by **FRIDAY 16TH AUGUST**.

 Based on our piloting, it should take between 45 minutes and one hour to complete.

 Please go to the next page for instructions on how to complete this survey.

| Page Break |  |
| --- | --- |

Instructions

You will be provided with statements which describe the potential elements of a new intervention for preschool children **(3:0 to 4:11 years)** with **co-occurring features** of developmental language disorder **(DLD)** and a speech sound disorder **(SSD)** *(phonological with consistent errors).*

The intervention is focusing on 1) **expressive vocabulary,** and 2) **speech comprehensibility/intelligibility** (i.e. how well the child can be understood by others in their everyday environments). The resulting description of this intervention will then be trialled for effectiveness in subsequent research.

The statements are broken down into **5 sections:** 1) **target setting** (11 statements), 2) **intervention content** (techniques and activities) (20 statements), 3) **intervention delivery** (8 statements), 4) **manual contents** (4 statements), and 5) **future adaptations** (4 statements). There are 47 statements for you to rate in total.

Please **rate each statement** and corresponding rationale from **1 (not at all appropriate)** to **5 (very appropriate)**. You have the **option to expand** on your response for each statement in the corresponding free text boxes. We particularly encourage this if statements are rated not at all appropriate, not appropriate, or neither appropriate nor inappropriate.


**Key**
The following acronyms will be used to show the original sources for each statement. Most statements have more than one source. You will be reminded of these acronyms on each page. 

 **EB.** The wider evidence base, including recent developments in the field of behaviour change.

 **S.** A nationwide (UK) survey of speech and language therapy practice (for targeting expressive vocabulary and speech comprehensibility within a combined intervention).

**SG.** Steering group input. The steering group consists of 3 speech and language therapists with relevant SSD/DLD specialisms (and one with equality, diversity and inclusion expertise), a specialist teacher, a parent of a child with SSD/DLD, an adult with DLD, and a bi/multilingual support worker (who collaborates with speech and language therapists).

 **SR**. A systematic review of intervention techniques for expressive vocabulary and speech comprehensibility.

| Page Break |  |
| --- | --- |

1.Target setting

***Reminder*** *Intervention group: 3:0-4:11 years, features of both DLD and a consistent phonological SSD, targeting expressive vocabulary + speech comprehensibility/intelligibility at the same time*
   *Key (sources where each statement came from)
 EB. The wider evidence base, including recent developments in the field of behaviour change.
 S. A nationwide (UK) survey of speech and language therapy practice (for targeting expressive vocabulary and speech comprehensibility within a combined intervention).
 SG. Steering group input (3 speech and language therapists with relevant SSD/DLD specialisms (and one with equality, diversity and inclusion expertise), a specialist teacher, a parent of a child with SSD/DLD, an adult with DLD, and a bi/multilingual support worker (who collaborates with speech and language therapists).
 SR. A systematic review of intervention techniques for expressive vocabulary and speech comprehensibility.*
  
**SECTION 1/5: Target setting (11 statements to rate)**
 
This section is about setting targets for the intervention, including: 

 1.  Intervention targets (the targets themselves)
 2.  Behaviour change techniques related to target setting (how parents are supported to set targets)
 3.  Wider considerations (additional key principles relating to the target setting)

Q1 target setting 1. Target one: The intervention will include a vocabulary target which is based on both developmental norms and words the child is most likely to need within daily life (S, SG).

*Rationale: this will facilitate a short-term functional impact for the child as well as aiding future language growth/sentence construction.*

- 1 Very inappropriate
- 2 Inappropriate
- 3 Neither appropriate or inappropriate
- 4 Appropriate
- 5 Very appropriate

Q1b 1b. Additional comments/justification of your selection

 *(optional- but recommended if you selected 'very inappropriate', 'inappropriate' or 'neither inappropriate or inappropriate')*

________________________________________________________________

________________________________________________________________

________________________________________________________________

________________________________________________________________

________________________________________________________________

Q2 Target setting 2. Target two: The intervention will include a phonological awareness target which is suitable for their developmental level (i.e. attention and listening capacity) (EB, S, SG).

 *Rationale: Phonology is a shared difficulty in pre-schoolers with this profile. Working on phonology has the potential to impact speech production and lays the groundwork for later literacy development.*

- 1 Very inappropriate
- 2 Inappropriate
- 3 Neither appropriate or inappropriate
- 4 Appropriate
- 5 Very appropriate

Q2b 2b. Additional comments/justification of your selection

 *(optional- but recommended if you selected 'very inappropriate', 'inappropriate' or 'neither inappropriate or inappropriate')*

________________________________________________________________

________________________________________________________________

________________________________________________________________

________________________________________________________________

________________________________________________________________

Q3 Target setting 3. Target three: The intervention will include a sound awareness target, based on the child’s speech process errors (EB, SG).

*Rationale: Consistent speech process errors are mislearnt sound patterns. By targeting a process, this mislearning can be challenged. It also lays the foundation for future direct work for speech if needed.*

- 1 Very inappropriate
- 2 Inappropriate
- 3 Neither appropriate or inappropriate
- 4 Appropriate
- 5 Very appropriate

Q3b 3b. Additional comments/justification of your selection

 *(optional- but recommended if you selected 'very inappropriate', 'inappropriate' or 'neither inappropriate or inappropriate')*

________________________________________________________________

________________________________________________________________

________________________________________________________________

________________________________________________________________

________________________________________________________________

Q4 Target setting 4. Target four: The intervention will include a comprehensibility target (e.g. use of aided language boards, telling the child “show me”), based on which activities their comprehensibility is most impacting (EB, S, SG).

 *Rationale: This will have an immediate positive impact on the child’s functional communication in everyday life, whilst their speech intelligibility is still developing.*

- 1 Very inappropriate
- 2 Inappropriate
- 3 Neither appropriate or inappropriate
- 4 Appropriate
- 5 Very appropriate

Q4b 4b. Additional comments/justification of your selection

 *(optional- but recommended if you selected 'very inappropriate', 'inappropriate' or 'neither inappropriate or inappropriate')*

________________________________________________________________

________________________________________________________________

________________________________________________________________

________________________________________________________________

________________________________________________________________

Q5 Target setting 5. Set behaviour goal BCT (behaviour change technique):
The parent and clinician will set goals for how the parent will carry out the intervention techniques (EB, SG).

 *Rationale: By doing this jointly, the SLT can make sure that the way in which the parent carries out the intervention techniques is both relevant and feasible for them.*

- 1 Very inappropriate
- 2 Inappropriate
- 3 Neither appropriate or inappropriate
- 4 Appropriate
- 5 Very appropriate

Q5b 5b. Additional comments/justification of your selection

 *(optional- but recommended if you selected 'very inappropriate', 'inappropriate' or 'neither inappropriate or inappropriate')*

________________________________________________________________

________________________________________________________________

________________________________________________________________

________________________________________________________________

________________________________________________________________

Q6 Target setting 6. Goal strategizing BCT:
The parent and clinician will talk through barriers to implementing techniques and review strategies for overcoming these barriers (EB, SG).

 *Rationale: By making potential barriers explicit, the SLT can problem solve with the parent how to overcome them, so that implementation is as accessible for the parent as possible.*

- 1 Very inappropriate
- 2 Inappropriate
- 3 Neither appropriate or inappropriate
- 4 Appropriate
- 5 Very appropriate

Q6b 6b. Additional comments/justification of your selection

 *(optional- but recommended if you selected 'very inappropriate', 'inappropriate' or 'neither inappropriate or inappropriate')*

________________________________________________________________

________________________________________________________________

________________________________________________________________

________________________________________________________________

________________________________________________________________

Q7 Target setting 7. Agree outcome goal BCT:
The parent and clinician will agree the child’s targets (EB, SG).

*Rationale: By agreeing the child’s targets, parents have more agency, and therefore will potentially have more motivation to carry out the targets at home. The targets are also more likely to be relevant to the child.*

- 1 Very inappropriate
- 2 Inappropriate
- 3 Neither appropriate or inappropriate
- 4 Appropriate
- 5 Very appropriate

Q7b 7b. Additional comments/justification of your selection

 *(optional- but recommended if you selected 'very inappropriate', 'inappropriate' or 'neither inappropriate or inappropriate')*

________________________________________________________________

________________________________________________________________

________________________________________________________________

________________________________________________________________

________________________________________________________________

Q8 Target setting 8. Action planning BCT:
The parent and clinician will plan when, where and how the intervention techniques will be carried out (EB, SG).

*Rationale: By having a clear, joint plan for implementation, implementation at home will be more feasible and accessible.*

- 1 Very inappropriate
- 2 Inappropriate
- 3 Neither appropriate or inappropriate
- 4 Appropriate
- 5 Very appropriate

Q8b 8b. Additional comments/justification of your selection

 *(optional- but recommended if you selected 'very inappropriate', 'inappropriate' or 'neither inappropriate or inappropriate')*

________________________________________________________________

________________________________________________________________

________________________________________________________________

________________________________________________________________

________________________________________________________________

Q9 Target setting 9. Where the child is bi/multilingual, intervention targets will align with any available norms for their home language (EB, SG)

 *Rationale: Different languages have different phonological inventories (sound patterns). It would be inappropriate to target an error process in the child’s home language if this does not typically develop until they are much older.*

- 1 Very inappropriate
- 2 Inappropriate
- 3 Neither appropriate or inappropriate
- 4 Appropriate
- 5 Very appropriate

Q9b 9b. Additional comments/justification of your selection

 *(optional- but recommended if you selected 'very inappropriate', 'inappropriate' or 'neither inappropriate or inappropriate')*

________________________________________________________________

Q10 Target setting 10. Before setting targets, the clinician will liaise with other services (where relevant) to ascertain prior and current support received (SG)

 *Rationale: By liaising with other services, the SLT can start “where the family is at” and make sure that the family is getting wider support they might be entitled to.*

- 1 Very inappropriate
- 2 Inappropriate
- 3 Neither appropriate or inappropriate
- 4 Appropriate
- 5 Very appropriate

Q10b 10b. Additional comments/justification of your selection

 *(optional- but recommended if you selected 'very inappropriate', 'inappropriate' or 'neither inappropriate or inappropriate')*

________________________________________________________________

________________________________________________________________

________________________________________________________________

________________________________________________________________

________________________________________________________________

Q11 Target setting 11. Intervention techniques and activities will be explicitly linked to the target they are addressing (SG).

 *Rationale: By having a clear link between targets and content, parents and wider support networks will better understand the purpose of what they are doing.*

- 1 Very inappropriate
- 2 Inappropriate
- 3 Neither appropriate or inappropriate
- 4 Appropriate
- 5 Very appropriate

Q11b 11b. Additional comments/justification of your selection

 *(optional- but recommended if you selected 'very inappropriate', 'inappropriate' or 'neither inappropriate or inappropriate')*

________________________________________________________________

| Page Break |  |
| --- | --- |

2.Content

***Reminder*** *Intervention group: 3:0-4:11 years, features of both DLD and a consistent phonological SSD, targeting expressive vocabulary + speech comprehensibility/intelligibility at the same time*
  
 *Key (sources where each statement came from)
 EB. The wider evidence base, including recent developments in the field of behaviour change.
 S. A nationwide (UK) survey of speech and language therapy practice (for targeting expressive vocabulary and speech comprehensibility within a combined intervention).
 SG. Steering group input (3 speech and language therapists with relevant SSD/DLD specialisms (and one with equality, diversity and inclusion expertise), a specialist teacher, a parent of a child with SSD/DLD, an adult with DLD, and a bi/multilingual support worker (who collaborates with speech and language therapists).
 SR. A systematic review of intervention techniques for expressive vocabulary and speech comprehensibility.*

 **SECTION 2/5: Intervention content (20 statements to rate)**

 This section is about the content for the intervention, including:

 1.Intervention techniques (techniques for each target area)
 2.Behaviour change techniques (how parents are supported to deliver content)
 3.Wider considerations (additional key principles relating to intervention content)

Q12 Content 12. Target one: Vocabulary will be targeted through adult exposure according to the child’s level of language development (e.g. single word modelling for minimally verbal children, match + one for early combiners) (EB, S, SG, SR).

*Rationale: Exposure in a variety of contexts and structures is in line with cross-situational learning principles for vocabulary development.*

- 1 Very inappropriate
- 2 Inappropriate
- 3 Neither appropriate or inappropriate
- 4 Appropriate
- 5 Very appropriate

Q12b 12b. Additional comments/justification of your selection

*(optional- but recommended if you selected 'very inappropriate', 'inappropriate' or 'neither inappropriate or inappropriate')*

________________________________________________________________

________________________________________________________________

________________________________________________________________

________________________________________________________________

________________________________________________________________

Q13 Content 13. Target two: Phonological awareness will be targeted through syllable segmentation activities, or word segmentation if the child is not ready for syllable work yet (EB, S, SG). 

*Rationale: Poor syllable segmentation, and persistent difficulties with polysyllabic words, is a key feature for children with this profile. These sentence and word level skills are foundational to future literacy development and lay the groundwork for more advanced phonological awareness work in the future.*

- 1 Very inappropriate
- 2 Inappropriate
- 3 Neither appropriate or inappropriate
- 4 Appropriate
- 5 Very appropriate

Q13b 13b. Additional comments/justification of your selection

 *(optional- but recommended if you selected 'very inappropriate', 'inappropriate' or 'neither inappropriate or inappropriate')*

________________________________________________________________

________________________________________________________________

________________________________________________________________

________________________________________________________________

________________________________________________________________

Q14 content 14. Target three: Sound awareness relating to error processes will be targeted through hybrid use of focused auditory stimulation (also known as auditory bombardment), recasting, visual cues (e.g. cued articulation), and exposure to word contrasts (EB, S, SG, SR).

*Rationale: Helpful techniques for developing sound awareness (of an error process) are inter-connected and can complement each other. Visual referents can reinforce the auditory processing of sounds.*

- 1 Very inappropriate
- 2 Inappropriate
- 3 Neither appropriate or inappropriate
- 4 Appropriate
- 5 Very appropriate

Q14b 14b. Additional comments/justification of your selection

 *(optional- but recommended if you selected 'very inappropriate', 'inappropriate' or 'neither inappropriate or inappropriate')*

________________________________________________________________

________________________________________________________________

________________________________________________________________

________________________________________________________________

________________________________________________________________

Q15 content 15. Target four: Speech comprehensibility will be targeted through integration of strategies into everyday activities- e.g. using aided language boards at home, telling the child “show me” (EB, S, SG).

*Rationale: Such integration is more likely to have an immediate, positive impact for the child. By focusing on the child’s everyday activities, these strategies are more likely to be relevant to the individual child and family.*

- 1 Very inappropriate
- 2 Inappropriate
- 3 Neither appropriate or inappropriate
- 4 Appropriate
- 5 Very appropriate

Q15b 15b. Additional comments/justification of your selection

*(optional- but recommended if you selected 'very inappropriate', 'inappropriate' or 'neither inappropriate or inappropriate')*

________________________________________________________________

Q16 content 16. Target four: Strategies to support with speech comprehensibility in everyday life (e.g. selecting vocabulary for picture boards) will be co-produced with the child’s family (SG).

*Rationale: By developing these strategies (and aids for delivering them) together, the SLT is better able to make sure that they are meaningful to the child and family and are culturally inclusive.*

- 1 Very inappropriate
- 2 Inappropriate
- 3 Neither appropriate or inappropriate
- 4 Appropriate
- 5 Very appropriate

Q16b 16b. Additional comments/justification of your selection

 *(optional- but recommended if you selected 'very inappropriate', 'inappropriate' or 'neither inappropriate or inappropriate')*

________________________________________________________________

________________________________________________________________

________________________________________________________________

________________________________________________________________

________________________________________________________________

Q17 content 17. Knowledge development behaviour BCT:
The Clinician will explain to the parent the rationale for intervention and what speech and language therapy is (EB, SG).

*Rationale: By giving parents this knowledge, this will empower them to more fully understand their child’s needs and make informed choices.*

- 1 Very inappropriate
- 2 Inappropriate
- 3 Neither appropriate or inappropriate
- 4 Appropriate
- 5 Very appropriate

Q17b 17b. Additional comments/justification of your selection

 *(optional- but recommended if you selected 'very inappropriate', 'inappropriate' or 'neither inappropriate or inappropriate')*

________________________________________________________________

________________________________________________________________

________________________________________________________________

________________________________________________________________

________________________________________________________________

Q18 content 18. Provide feedback BCT (behaviour change technique):
 The clinician will give feedback to the parent about how they are conducting intervention techniques (EB, SG, SR).

*Rationale: Feedback will help the parent to maintain and improve on their technique implementation.*

- 1 Very inappropriate
- 2 Inappropriate
- 3 Neither appropriate or inappropriate
- 4 Appropriate
- 5 Very appropriate

Q18b 18b. Additional comments/justification of your selection

*(optional- but recommended if you selected 'very inappropriate', 'inappropriate' or 'neither inappropriate or inappropriate')*

________________________________________________________________

________________________________________________________________

________________________________________________________________

________________________________________________________________

________________________________________________________________

Q19 content 19. Provide feedback on outcome of behaviour BCT:
 The clinician will give feedback to the parent on the impact of them conducting the intervention techniques (i.e. the change observed in the child) (EB, SG).

 *Rationale: By drawing attention to the positive consequences on the child of the parent carrying out the techniques, parents will feel more encouraged and this could support motivation.*

- 1 Very inappropriate
- 2 Inappropriate
- 3 Neither appropriate or inappropriate
- 4 Appropriate
- 5 Very appropriate

Q19b 19b. Additional comments/justification of your selection

 *(optional- but recommended if you selected 'very inappropriate', 'inappropriate' or 'neither inappropriate or inappropriate')*

________________________________________________________________

________________________________________________________________

________________________________________________________________

________________________________________________________________

________________________________________________________________

Q20 content 20. Self-monitor behaviour BCT :
The parent will monitor how they are continuing with techniques/activities at home as an informal measure of progress (EB, SG).

*Rationale: By monitoring how they are getting on the parent will be able to modify their input accordingly. This will also help them to maintain independence with technique use between sessions with the SLT.*

- 1 Very inappropriate
- 2 Inappropriate
- 3 Neither appropriate or inappropriate
- 4 Appropriate
- 5 Very appropriate

Q20b 20b. Additional comments/justification of your selection

 *(optional- but recommended if you selected 'very inappropriate', 'inappropriate' or 'neither inappropriate or inappropriate')*

________________________________________________________________

________________________________________________________________

________________________________________________________________

________________________________________________________________

________________________________________________________________

Q21 content 21. Self-monitor outcome of behaviour BCT:
The parent will monitor the impact of their work with their child at home as an informal measure of progress (EB, SG).

*Rationale: By seeing potential positive impacts of them delivering intervention techniques with their child, parents may feel more encouraged and motivated. Additionally, this will empower them to monitor their child’s speech and language development when their child is not receiving therapy.*

- 1 Very inappropriate
- 2 Inappropriate
- 3 Neither appropriate or inappropriate
- 4 Appropriate
- 5 Very appropriate

Q21b 21b. Additional comments/justification of your selection

 *(optional- but recommended if you selected 'very inappropriate', 'inappropriate' or 'neither inappropriate or inappropriate')*

________________________________________________________________

________________________________________________________________

________________________________________________________________

________________________________________________________________

________________________________________________________________

Q22 content 22. Social Support BCT :
The clinician will take the time with the parent to establish who is best placed to deliver the intervention techniques, and wider family support which might facilitate intervention implementation. (EB, SG)

*Rationale: Wider family networks and friends may be able to facilitate the carrying out of techniques and provide support for the parent at home.*

- 1 Very inappropriate
- 2 Inappropriate
- 3 Neither appropriate or inappropriate
- 4 Appropriate
- 5 Very appropriate

Q22b 22b. Additional comments/justification of your selection

 *(optional- but recommended if you selected 'very inappropriate', 'inappropriate' or 'neither inappropriate or inappropriate')*

________________________________________________________________

________________________________________________________________

________________________________________________________________

________________________________________________________________

________________________________________________________________

Q23 content 23. Inform about social consequences BCT:
The clinician will talk to the parent about the potential social consequences (positive or negative) of carrying out the intervention techniques (EB, SG). *Note: positives emphasised, negatives kept to a minimum and to be discussed sensitively.*

*Rationale: The parent understands why the intervention and related techniques are important. This may support with motivation.*

- 1 Very inappropriate
- 2 Inappropriate
- 3 Neither appropriate or inappropriate
- 4 Appropriate
- 5 Very appropriate

Q23b 23b. Additional comments/justification of your selection

 *(optional- but recommended if you selected 'very inappropriate', 'inappropriate' or 'neither inappropriate or inappropriate')*

________________________________________________________________

________________________________________________________________

________________________________________________________________

________________________________________________________________

________________________________________________________________

Q24 content 24. Inform about environmental consequences BCT:
The clinician will talk to the parent about the potential environmental consequences (positive or negative) of carrying out the intervention techniques (only IF the parent is in a position to act on this information) (EB, SG). *Note: positives emphasised, negatives kept to a minimum and to be discussed sensitively*

*Rationale: The parent understands why the intervention and related techniques are important. This may support with motivation.*

- 1 Very inappropriate
- 2 Inappropriate
- 3 Neither appropriate or inappropriate
- 4 Appropriate
- 5 Very appropriate

Q24b 24b. Additional comments/justification of your selection

 *(optional- but recommended if you selected 'very inappropriate', 'inappropriate' or 'neither inappropriate or inappropriate')*

________________________________________________________________

________________________________________________________________

________________________________________________________________

________________________________________________________________

________________________________________________________________

Q25 content 25. Demonstrate the behaviour BCT :
The clinician will model techniques for the parent to see (EB, SG).

*Rationale: The parent is more likely to learn the technique if given a tangible example in person.*

- 1 Very inappropriate
- 2 Inappropriate
- 3 Neither appropriate or inappropriate
- 4 Appropriate
- 5 Very appropriate

Q25b 25b. Additional comments/justification of your selection

 *(optional- but recommended if you selected 'very inappropriate', 'inappropriate' or 'neither inappropriate or inappropriate')*

________________________________________________________________

________________________________________________________________

________________________________________________________________

________________________________________________________________

________________________________________________________________

Q26 content 26. Reduce cue frequency BCT:
The clinician will gradually withdraw prompting/cues when the parent is carrying out the technique (EB, SG).

*Rationale: Withdrawing cues will enable to parent to gradually become more independent when carrying out the intervention techniques.*

- 1 Very inappropriate
- 2 Inappropriate
- 3 Neither appropriate or inappropriate
- 4 Appropriate
- 5 Very appropriate

Q26b 26b. Additional comments/justification of your selection

*(optional- but recommended if you selected 'very inappropriate', 'inappropriate' or 'neither inappropriate or inappropriate')*

________________________________________________________________

________________________________________________________________

________________________________________________________________

________________________________________________________________

________________________________________________________________

Q27 content 27. Skill development behaviour BCT:
 The parent will practice carrying out the technique with the clinician (EB, SG).

*Rationale: The parent is more likely to learn the technique if they get hands on practice with a clinician guiding them.*

- 1 Very inappropriate
- 2 Inappropriate
- 3 Neither appropriate or inappropriate
- 4 Appropriate
- 5 Very appropriate

Q27b 27b. Additional comments/justification of your selection

 *(optional- but recommended if you selected 'very inappropriate', 'inappropriate' or 'neither inappropriate or inappropriate')*

________________________________________________________________

________________________________________________________________

________________________________________________________________

________________________________________________________________

________________________________________________________________

Q28 content 28. Provide positive social consequence for the behaviour BCT :
The clinician will provide praise when the parent is making progress with implementing the techniques (EB, SG).
  *Rationale: As well as highlighting to the parent what they should continue doing, this will make the experience more positive for the parent and support with motivation.*

- 1 Very inappropriate
- 2 Inappropriate
- 3 Neither appropriate or inappropriate
- 4 Appropriate
- 5 Very appropriate

Q28b 28b. Additional comments/justification of your selection

 *(optional- but recommended if you selected 'very inappropriate', 'inappropriate' or 'neither inappropriate or inappropriate')*

________________________________________________________________

Q29 content 29. Techniques will be primarily input based, with flexibility to elicit speech/language directly from the child if they demonstrate readiness for this (S, SG).
  *Rationale: The child may lack confidence or have difficulties sustaining their attention. Heavily encouraging the child to produce speech/language if they are not ready may have negative consequences for their future involvement in speech and language therapy.*

- 1 Very inappropriate
- 2 Inappropriate
- 3 Neither appropriate or inappropriate
- 4 Appropriate
- 5 Very appropriate

Q29b 29b. Additional comments/justification of your selection

 *(optional- but recommended if you selected 'very inappropriate', 'inappropriate' or 'neither inappropriate or inappropriate')*

________________________________________________________________

________________________________________________________________

________________________________________________________________

________________________________________________________________

________________________________________________________________

Q30 content 30. ‘Readiness’ for direct elicitation of speech/language will be jointly decided by the clinician and family (and child where possible) (SG).

 *Rationale: The parent knows the child best and can give insight into how the child has responded to direct elicitation in the past.*

- 1 Very inappropriate
- 2 Inappropriate
- 3 Neither appropriate or inappropriate
- 4 Appropriate
- 5 Very appropriate

Q30b 30b. Additional comments/justification of your selection

 *(optional- but recommended if you selected 'very inappropriate', 'inappropriate' or 'neither inappropriate or inappropriate')*

________________________________________________________________

Q31 content 31. The intervention will include a flexible option of activities/routines for parents to incorporate relevant language techniques into, with support to identify their own (SG).

 *Rationale: By enabling parents to choose the best activities/routines to integrate the language techniques into, they are more likely to be culturally inclusive, reflect the child’s current preferences, and feasible for implementation at home.*

- 1 Very inappropriate
- 2 Inappropriate
- 3 Neither appropriate or inappropriate
- 4 Appropriate
- 5 Very appropriate

Q31b 31b. Additional comments/justification of your selection

 *(optional- but recommended if you selected 'very inappropriate', 'inappropriate' or 'neither inappropriate or inappropriate')*

________________________________________________________________

________________________________________________________________

________________________________________________________________

________________________________________________________________

________________________________________________________________

| Page Break |  |
| --- | --- |

3.Delivery

***Reminder*** *Intervention group: 3:0-4:11 years, features of both DLD and a consistent phonological SSD, targeting expressive vocabulary + speech comprehensibility/intelligibility at the same time*
  
 *Key (sources where each statement came from)
 EB. The wider evidence base, including recent developments in the field of behaviour change.
 S. A nationwide (UK) survey of speech and language therapy practice (for targeting expressive vocabulary and speech comprehensibility within a combined intervention).
 SG. Steering group input (3 speech and language therapists with relevant SSD/DLD specialisms (and one with equality, diversity and inclusion expertise), a specialist teacher, a parent of a child with SSD/DLD, an adult with DLD, and a bi/multilingual support worker (who collaborates with speech and language therapists).
 SR. A systematic review of intervention techniques for expressive vocabulary and speech comprehensibility.*

 **SECTION 3/5: Intervention delivery (8 statements to rate)**

 This section is about the delivery of the intervention, including:

 1. Who, where and dosage
 2. Behaviour change techniques related to delivery (i.e. how best to support parents with delivery)

Q32 delivery 32. The speech and phonological awareness aspects of the intervention will be primarily managed and delivered by the clinician in clinic* (EB, SG, SR).

*Rationale: Added burden for the parent if they are expected to work on speech and language at home (time commitment). By working on speech/phonological awareness in clinic, the SLT is able to control for exposure to items with particular sounds and use their expertise to modify their input in response to the child’s productions.

 *Later in this survey, you will be given the opportunity to rate the appropriateness of the intervention being adapted in the future for delivery solely within the child’s home*

- 1 Very inappropriate
- 2 Inappropriate
- 3 Neither appropriate or inappropriate
- 4 Appropriate
- 5 Very appropriate

Q32b 32b. Additional comments/justification of your selection

 *(optional- but recommended if you selected 'very inappropriate', 'inappropriate' or 'neither inappropriate or inappropriate')*

________________________________________________________________

________________________________________________________________

________________________________________________________________

________________________________________________________________

________________________________________________________________

Q33 delivery 33. The language aspects of the intervention will primarily be delivered through the supported parent using language facilitation techniques in the child’s everyday life* (EB, S, SG, SR).

*Rationale: Multiple exposures to language in meaningful contexts for the child will facilitate child engagement and wider generalisation of language learnt. The parent knows their child best and can implement techniques in multiple relevant contexts as they see fit (e.g. bathtime, at the park, on holiday, visiting the dentist).

 *Later in the survey, you will be given the opportunity to rate the appropriateness of the intervention being adapted in the future for delivery via educational settings*

- 1 Very inappropriate
- 2 Inappropriate
- 3 Neither appropriate or inappropriate
- 4 Appropriate
- 5 Very appropriate

Q33b 33b. Additional comments/justification of your selection

*(optional- but recommended if you selected 'very inappropriate', 'inappropriate' or 'neither inappropriate or inappropriate')*

________________________________________________________________

________________________________________________________________

________________________________________________________________

________________________________________________________________

________________________________________________________________

Q34 delivery 34. Guidance will be given regarding dosage ranges for intervention techniques (technique dosage per activity) (EB, S, SG).

*Rationale: Having an approximate idea of how many times a technique should be used in an activity will enable both the parent and SLT to know what they are aiming for, and what is most likely to be effective.*

- 1 Very inappropriate
- 2 Inappropriate
- 3 Neither appropriate or inappropriate
- 4 Appropriate
- 5 Very appropriate

Q34b 34b. Additional comments/justification of your selection

*(optional- but recommended if you selected 'very inappropriate', 'inappropriate' or 'neither inappropriate or inappropriate')*

________________________________________________________________

________________________________________________________________

________________________________________________________________

________________________________________________________________

________________________________________________________________

Q35 delivery 35. Guidance will be given regarding an intervention duration (including number of sessions and spacing of sessions) (EB, S, SG, SR)

*Rationale: Having a duration for the intervention will enable the SLT to plan accordingly and help the parent to know what to expect from the outset.*

- 1 Very inappropriate
- 2 Inappropriate
- 3 Neither appropriate or inappropriate
- 4 Appropriate
- 5 Very appropriate

Q35b 35b. Additional comments/justification of your selection

*(optional- but recommended if you selected 'very inappropriate', 'inappropriate' or 'neither inappropriate or inappropriate')*

________________________________________________________________

Q36 delivery 36. If the intervention is being delivered with an interpreter, at least double time should be allocated (EB, SG).

*Rationale: Sessions with interpreters are more time consuming. It is discrimination if an interpreter is needed but no additional time is provided.*

- 1 Very inappropriate
- 2 Inappropriate
- 3 Neither appropriate or inappropriate
- 4 Appropriate
- 5 Very appropriate

Q36b 36b. Additional comments/justification of your selection

*(optional- but recommended if you selected 'very inappropriate', 'inappropriate' or 'neither inappropriate or inappropriate')*

________________________________________________________________

________________________________________________________________

________________________________________________________________

________________________________________________________________

________________________________________________________________

Q37 delivery 37. Intervention activities should be changed if the child lacks motivation or is not enjoying them (S, SG).

*Rationale: If the child is not enjoying the activity they are more likely to lose attention; this will have a knock-on effect on their subsequent language learning.*

- 1 Very inappropriate
- 2 Inappropriate
- 3 Neither appropriate or inappropriate
- 4 Appropriate
- 5 Very appropriate

Q37b 37b. Additional comments/justification of your selection

*(optional- but recommended if you selected 'very inappropriate', 'inappropriate' or 'neither inappropriate or inappropriate')*

________________________________________________________________

________________________________________________________________

________________________________________________________________

________________________________________________________________

________________________________________________________________

Q38 delivery 38. Add objects to the environment BCT:
 Parents to bring the child’s favourite toys/books/items from home into the clinic (EB, SG).

*Rationale: By having toys/objects from home brought in the child may feel more comfortable. It would also allow the SLT to use them when demonstrating language techniques during the session.*

- 1 Very inappropriate
- 2 Inappropriate
- 3 Neither appropriate or inappropriate
- 4 Appropriate
- 5 Very appropriate

Q38b 38b. Additional comments/justification of your selection

 *(optional- but recommended if you selected 'very inappropriate', 'inappropriate' or 'neither inappropriate or inappropriate')*

________________________________________________________________

________________________________________________________________

________________________________________________________________

________________________________________________________________

________________________________________________________________

Q39 delivery 39. Generalisation in learning BCT:
 Parents will be asked to deliver the intervention techniques from clinic at home (EB, SG).

*Rationale: By generalising their use of language techniques at home, the child will be supported in developing their language within meaningful environments.*

- 1 Very inappropriate
- 2 Inappropriate
- 3 Neither appropriate or inappropriate
- 4 Appropriate
- 5 Very appropriate

Q39b 39b. Additional comments/justification of your selection

 *(optional- but recommended if you selected 'very inappropriate', 'inappropriate' or 'neither inappropriate or inappropriate')*

________________________________________________________________

________________________________________________________________

________________________________________________________________

________________________________________________________________

________________________________________________________________

| Page Break |  |
| --- | --- |

4. Manual contents

***Reminder*** *Intervention group: 3:0-4:11 years, features of both DLD and a consistent phonological SSD, targeting expressive vocabulary + speech comprehensibility/intelligibility at the same time*
  
 *Key (sources where each statement came from)
 EB. The wider evidence base, including recent developments in the field of behaviour change.
 S. A nationwide (UK) survey of speech and language therapy practice (for targeting expressive vocabulary and speech comprehensibility within a combined intervention).
 SG. Steering group input (3 speech and language therapists with relevant SSD/DLD specialisms (and one with equality, diversity and inclusion expertise), a specialist teacher, a parent of a child with SSD/DLD, an adult with DLD, and a bi/multilingual support worker (who collaborates with speech and language therapists).
 SR. A systematic review of intervention techniques for expressive vocabulary and speech comprehensibility.*
  
 **SECTION 4/5: Manual contents (4 statements to rate)**

 The intervention will include an intervention manual for clinicians. This should provide the necessary information for them to carry out the intervention. These questions are about what goes into this manual. 

Q40 manual 40. The intervention manual will include a flexible discussion guide to support both the clinician in getting to know the family, and the family in understanding more about speech and language therapy (EB, SG).

*Rationale: Understanding the child and their family is the foundation for what comes next. Important for the SLT to develop a positive rapport with the parent from the outset and have an understanding of what barriers (and supports) they have in everyday life.*

- 1 Very inappropriate
- 2 Inappropriate
- 3 Neither appropriate or inappropriate
- 4 Appropriate
- 5 Very appropriate

Q40b 40b. Additional comments/justification of your selection

________________________________________________________________

________________________________________________________________

________________________________________________________________

________________________________________________________________

________________________________________________________________

Q41 manual 41. The intervention manual will include guidance on what ‘readiness’ for direct speech work (i.e. eliciting words from the child) could look like (S, SG).

*Rationale: Definition of ‘readiness’, and what this looks like in practice, may vary (e.g. is it to do with confidence, attention, or both?).*

- 1 Very inappropriate
- 2 Inappropriate
- 3 Neither appropriate or inappropriate
- 4 Appropriate
- 5 Very appropriate

Q41b 41b. Additional comments/justification of your selection

 *(optional- but recommended if you selected 'very inappropriate', 'inappropriate' or 'neither inappropriate or inappropriate')*

________________________________________________________________

________________________________________________________________

________________________________________________________________

________________________________________________________________

________________________________________________________________

Q42 manual 42. The intervention manual will include targets which can be selected across the 4 target areas, as well as guidance around progression and links to relevant non-English speech/language norm resources (SG).

*Rationale: Targets chosen from the manual can be explicitly linked to the intervention content. Children often progress at different rates. Guidance around progression will support the SLT in deciding where to go next if the child has made a lot of progress in relatively few sessions (or conversely, very little progress).*

- 1 Very inappropriate
- 2 Inappropriate
- 3 Neither appropriate or inappropriate
- 4 Appropriate
- 5 Very appropriate

Q42b 42b. Additional comments/justification of your selection

 *(optional- but recommended if you selected 'very inappropriate', 'inappropriate' or 'neither inappropriate or inappropriate')*

________________________________________________________________

________________________________________________________________

________________________________________________________________

________________________________________________________________

________________________________________________________________

Q43 manual 43. The intervention manual will include key handouts for parents, which can be personalised (S, SG)

*Rationale: Having something to take away from the session will support the parent in remembering what was covered. By being able to personalise the handouts, they will be more relevant to that specific child and family.*

- 1 Very inappropriate
- 2 Inappropriate
- 3 Neither appropriate or inappropriate
- 4 Appropriate
- 5 Very appropriate

Q43b 43b. Additional comments/justification of your selection

 *(optional- but recommended if you selected 'very inappropriate', 'inappropriate' or 'neither inappropriate or inappropriate')*

________________________________________________________________

| Page Break |  |
| --- | --- |

5.Future adaptation

***Reminder*** *Intervention group: 3:0-4:11 years, features of both DLD and a consistent phonological SSD, targeting expressive vocabulary + speech comprehensibility/intelligibility at the same time*
  
 *Key (sources where each statement came from)
 EB. The wider evidence base, including recent developments in the field of behaviour change.
 S. A nationwide (UK) survey of speech and language therapy practice (for targeting expressive vocabulary and speech comprehensibility within a combined intervention).
 SG. Steering group input (3 speech and language therapists with relevant SSD/DLD specialisms (and one with equality, diversity and inclusion expertise), a specialist teacher, a parent of a child with SSD/DLD, an adult with DLD, and a bi/multilingual support worker (who collaborates with speech and language therapists).
 SR. A systematic review of intervention techniques for expressive vocabulary and speech comprehensibility.*
  
 **SECTION 5/5: Future adaptations (4 statements to rate)**

We need to keep certain factors **consistent** (e.g. deliverer, place of delivery) when first **trialling** the intervention. However, if effectiveness is indicated after initial trialling, the intervention might then be **adapted** to provide further 'reach'.

These statements are about possibilities for how the intervention might be adapted **in the future**.

 No rationale is given as we would encourage you to provide us with **a rationale for your choice** within the free text comments.

 Please note: each statement should be considered **on its own**, rather than in the context of other statements.

Q44 future 44. Option for the intervention to be delivered with education professionals, in educational settings (S, SG).

- 1 Very inappropriate
- 2 Inappropriate
- 3 Neither appropriate or inappropriate
- 4 Appropriate
- 5 Very appropriate

Q44b 44b. Additional comments/justification of your selection *(optional but encouraged)*

________________________________________________________________

Q45 future 45. Option for the intervention to be delivered both through face to face and online sessions (hybrid format) (EB, SG). 

- 1 Very inappropriate
- 2 Inappropriate
- 3 Neither appropriate or inappropriate
- 4 Appropriate
- 5 Very appropriate

Q45b 45b. Additional comments/justification of your selection *(optional but encouraged)*

________________________________________________________________

________________________________________________________________

________________________________________________________________

________________________________________________________________

________________________________________________________________

Q46 future 46. Option for full delivery at home, with the clinician doing home visits (SG).

- 1 Very inappropriate
- 2 Inappropriate
- 3 Neither appropriate or inappropriate
- 4 Appropriate
- 5 Very appropriate

Q46b 46b. Additional comments/justification of your selection *(optional but encouraged)*

________________________________________________________________

________________________________________________________________

________________________________________________________________

________________________________________________________________

________________________________________________________________

Q47 future 47. Future development of a shared ‘app’ for parents and clinicians as a tool for guiding intervention delivery and monitoring progress (EB, SG).

- 1 Very inappropriate
- 2 Inappropriate
- 3 Neither appropriate or inappropriate
- 4 Appropriate
- 5 Very appropriate

Q47b 47b. Additional comments/justification of your selection *(optional but encouraged)*

________________________________________________________________

________________________________________________________________

________________________________________________________________

________________________________________________________________

________________________________________________________________

| Page Break |  |
| --- | --- |

Feedback 48. Is there anything you would like to add or feedback you wish to give about this project or e-delphi in general?

________________________________________________________________

________________________________________________________________

________________________________________________________________

________________________________________________________________

________________________________________________________________

Nearly there! please click the submit arrow below to submit your responses

End of Block: Default Question Block
