# Supplementary material for: Co-design to consensus: Identifying the core elements of a novel intervention for pre-school children with co-occurring phonological speech sound disorder (SSD) and developmental language disorder (DLD) using a modified e-Delphi approach
Source: PLoS One. 2025 Jun 18;20(6):e0326072. doi: 10.1371/journal.pone.0326072 (PMC12176183; doi:10.1371/journal.pone.0326072)
Supplement: S7 — (DOCX) [file pone.0326072.s007.docx]

**S7: Reworded statements**

| Original statement | Reworded statement | Reason for re-wording |
| --- | --- | --- |
| Intervention techniques and activities will be explicitly linked to the target they are addressing. | Intervention techniques and activities will be explicitly linked to the target they are addressing. For example, explicitly stating that language modelling is to help the child achieve their language target. | Example needed |
| Inform about social consequences BCT: The clinician will talk to the parent about the potential social consequences (positive or negative) of carrying out the intervention techniques. Note: positives emphasised, negatives kept to a minimum and to be discussed sensitively. | Inform about social consequences BCT (behaviour change technique): The clinician will talk to the parent about the potential social consequences (positive or negative*) of carrying out the intervention techniques. For example, that an increase in language (as a result of carrying out a technique such as modelling) could help them to interact with their peers with more ease. | Example needed |
| If the intervention is being delivered with an interpreter, at least double time should be allocated | If the intervention is being delivered through an interpreter, double time should be allocated (this time might be spread across sessions, rather than the child having to attend one long session) | Consensus not reached *(75% agreement; median 5, IQR 3.25-5)*  Also asked to re-rate based on what they think, rather than what their service can currently offer. |
| Option for the intervention to be delivered both through face-to-face and online sessions (hybrid format) | In the future, there would be an option for the intervention to be delivered in a hybrid face-to-face and online format. Online sessions might focus on parent coaching, with face-to-face sessions consisting of direct work with the child. | Consensus not reached *(72.2% agreement; median 4, IQR 3-4)*  Also asked to re-rate based on what they think, rather than what their service can currently offer. |
| The speech and phonological awareness aspects of the intervention will be primarily managed and delivered by the clinician in clinic | The speech/ psycholinguistic aspects of the intervention will be primarily managed and delivered by the clinician in clinic, with the flexibility for parents to carry out simple support strategies at home if feasible (e.g. being taught a small number of cued articulation signs to use whilst language modelling). | Consensus not reached *(66.7% agreement; median 4, IQR 3-5)*  Also asked to re-rate based on what they think, rather than what their service can currently offer. |
| Option for full delivery at home, with the clinician doing home visits | In the future, there would be an option for the intervention to be delivered via the clinician doing home visits (with the parent conducting carryover activities between visits). | Consensus not reached *(66.7% agreement; median 4, IQR 3-5)*  Also asked to re-rate based on what they think, rather than what their service can currently offer. |
| The intervention will include a phonological awareness target which is suitable for their developmental level (i.e. attention and listening capacity). | The intervention will include a psycholinguistic target based on the child's developmental level (including attention and listening and phonological awareness ability), and speech error characteristics. | Merged into one statement |
| The intervention will include a sound awareness target, based on the child’s speech process errors. |  |  |
| Phonological awareness will be targeted through syllable segmentation activities, or word segmentation if the child is not ready for syllable work yet. | Depending on the child's developmental level, their speech sound system (i.e. psycholinguistic profile) will be supported through syllable/phoneme level awareness activities. These activities might integrate exposure to sound/word contrasts using techniques such as auditory stimulation (also known as auditory bombardment), recasting and visual cues (e.g. cued articulation | Merged into one statement |
| Sound awareness relating to error processes will be targeted through hybrid use of focused auditory stimulation (also known as auditory bombardment), recasting, visual cues (e.g. cued articulation), and exposure to word contrasts |  |  |
