# Supplementary material for: Co-design to consensus: Identifying the core elements of a novel intervention for pre-school children with co-occurring phonological speech sound disorder (SSD) and developmental language disorder (DLD) using a modified e-Delphi approach
Source: PLoS One. 2025 Jun 18;20(6):e0326072. doi: 10.1371/journal.pone.0326072 (PMC12176183; doi:10.1371/journal.pone.0326072)
Supplement: S8 — (DOCX) [file pone.0326072.s008.docx]

**S7 Interim feedback document**

Firstly- **THANK YOU** all for completing the first round. Before round 2 goes live, we would like to share the round 1 results with you.

To make this as accessible as possible we have not included all data (this is a separate 50+ page document). If you would like to see the full document, please email me at [lucy.rodgers@city.ac.uk](mailto:lucy.rodgers@city.ac.uk)

Reminder:

We are developing a novel intervention for pre-school children (3:0-4:11 years) with co-occurring SSD/DLD features, where expressive vocabulary and speech comprehensibility are joint outcomes.

The aim of this consensus work is to establish the core elements of the intervention. **We will be developing a detailed intervention protocol, based on the core elements agreed in this delphi, in the next phase of this intervention development work**. Your insightful free text responses containing such detail will therefore be integrated into the next phase.

We plan to email you the link for **round 2** by mid-September.


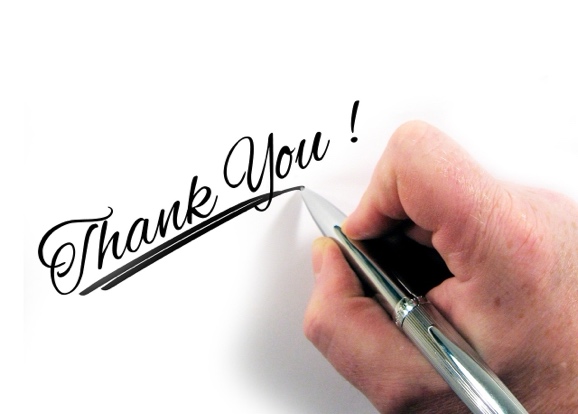


**Section 1: Overview**

We presented you **(36 panellists)** with **47 statements** in round 1.

**42 statements achieved consensus**. Meaning- they were rated as appropriate or very appropriate by 75% + of the group AND had an interquartile range of 1 or less. *(the interquartile range reflects the spread of responses between the 25% and 75% centile of the group. If it is 1 or under, it means that an acceptable proportion of responses fell within a relatively small range).*

There are **5 statements** which **did not achieve consensus**. All of these met our **minimum requirement of 50%** to be retained for round 2. Four of these statements will be reworded in view of your comments, and you will be given another opportunity to rate them in round 2 (*except for statement 24).* These statements are given in [**section 2**](#Section2).

There were **2 statements** which reached consensus where one or more panellists commented that they **did not fully understand** the wording. These statements will be re-worded, and you will be asked to rate them again in round 2. These statements are given in [**section 3**](#Section3).

There were some concerns about the number of targets and the difference between target 2 (phonological awareness) and target 3 (sound awareness). We have consulted with Helen Stringer (lead author for the RCSLT SSD guidelines) because of this and plan to propose a single, amended target in round 2 of the delphi. Your comments which prompted this are given in **[section 4](#Section4AGAIN)**[.](#Section4AGAIN)

The statistics for the statements that did achieve consensus are given in [**section 5**](#Section5).

**Section 2: Statements that did not achieve consensus**

***(4 of these will be amended for you to re-rate in round 2)***

Statement 24: Inform about environmental consequences BCT: The clinician will talk to the parent about the potential environmental consequences (positive or negative) of carrying out the intervention techniques (only IF the parent is in a position to act on this information) (EB, SG). Note: positives emphasised, negatives kept to a minimum and to be discussed sensitively. *Rationale: The parent understands why the intervention and related techniques are important. This may support with motivation.*

Percentage rated as appropriate or very appropriate: 75% (27/36)

Median and interquartile range: 4 (3.25/5)

Comments:

*I think if you are going to do this, you need to be completely honest about the positives and negatives otherwise it is misleading (panellist rated 2)*

*important to have clear expectations before starting intervention; plus to problem solve around barriers (panellist rated 4)*

***I'm not sure what is meant by environmental consequences (panellist rated 3)***

***Not sure what this means (panellist rated 3)***

***not sure what you mean (panellist rated 3)***

Note: Following your ratings and comments, which prompted a consultation with Dr Sarah Barnett *(author of the paper our Behaviour Change Technique (BCT) statements were derived from),* we will exclude this statement. Sarah clarified that for the purpose of this statement ‘environment‘ is more to do with factors such as air pollution, and it is the ‘social consequences’ (statement 23) which are specific to SLT.

Statement 32: *The speech and phonological awareness aspects of the intervention will be primarily managed and delivered by the clinician in clinic* (EB, SG, SR). Rationale: Added burden for the parent if they are expected to work on speech and language at home (time commitment). By working on speech/phonological awareness in clinic, the SLT is able to control for exposure to items with particular sounds and use their expertise to modify their input in response to the child’s productions.*

Percentage rated as appropriate or very appropriate: 66.7% (24/36)

Median and interquartile range: 4 (3/5)

Comments:

*This depends on the child and their comfort and rapport with clinician. Also depends on service ability to provide and parental ability to attend the necessary intensity for intervention (panellist rated 3)*

*Guidance from the clinician and modelling, but each session incorporating the parent and a focus on what the parent is going to be trying at home (panellist rated 2)*

*I think this very much depends on the parent (panellist rated 3)*

*This may be the ideal but realistically weekly therapy sessions will have minimal impact if not supported at home/in nursery (panellist rated 4)*

*Also avoids needing th check / train parents in PA skills being worked on - some don't get it (panellist rated 5)*

*I think intervention is likely to have more impact if parents carryover activities at home - I'm not sure why we wouldn't expect them to support with the PA and speech sound work. Clinicians are very unlikely to be able to see children in clinic with the frequency required for therapy to be effective without follow-up at home. (panellist rated 2)*

*I think yes for speech but for PA parents can do these activities at home and from my experience do them well. eg clapping syllables, looking for rhymes (panellist rated 3)*

*phonological awareness may be the foundation to difficulties experiences. Global phonological awareness activities that aren't focusing on error patterns (e.g., syllable segmentation and deletion) can increase efficacy of speech sound intervention and are relatively easy for parents to carry out incidentally (panellist rated 1)*

*This should also be something completed at home at times, just not as the main aim. If phonological awareness is having the primary impact on clarity with some words then why not be able to segment this at home too (panellist rated 3)*

*Vulnerable families may be easier to engage with if home visits are offered. But strongly agree that speech and phonological awareness should be managed by the SLT.(panellist rated 3)*

*The frequency and dosage for the child would be insufficient to effect change; families need to be supported to engage with these targets. Phonological awareness tasks can be very simple and straightforward for parents to support; language strategies are less 'visible' to parents (panellist rated 2)*

*Think the auditory bombardment could be done by parents after watching sessions done by therapist. (panellist rated 3)*

*initially but with the aim of enskilling others to also support. (panellist rated 4)*

*although capacity within the NHS means that this cannot always happen (panellist rated 4)*

*Could some fun based phonological awareness tasks also be given to parents / carers? For example syllable segmentation modelling and activities in everyday situations (panellist rated 4)*

*I do not tend to address speech and language in one therapy block. I would focus on language (with the expectation that parents will carry out supports e.g. ACI strategies at home) and speech/ phonological awareness once language supports are in place (again with the expectation that parents regularly carry out activities at home). (panellist rated 3)*

*Not sure about this - would hope to include easily achievable activities and targets so that parent didn't feel 'over-burdoned' (eg Phon Awareness activities could be included with some of the vocab work) (panellist rated 3)*

*But this is so highly dependent on the individual child, it is difficult to plan a delivery as a 'one size fits all' as some children will struggle with clinic delivery, others will cooperate with clinician in clinic, but not in the home setting (panellist rated 4)*

Statement 36: If the intervention is being delivered with an interpreter, at least double time should be allocated (EB, SG). *Rationale: Sessions with interpreters are more time consuming. It is discrimination if an interpreter is needed but no additional time is provided.*

Percentage rated as appropriate or very appropriate: 75% (27/36)

Median and interquartile range: 5 (3.25/5)

Comments:

*Double time may not be needed but clinician should allow for a longer session (panellist rated 3)*

*This is likely to be dependent on resources available for therapist (time and financial cost) (panellist rated 3)*

*Ideally this is very appropriate, may be difficult with service restrictions (panellist rated 5)*

*This isn't my area, but certainly there will need to be training and practice with the interpreter prior to working alongside the child, as well as the added demands of each language (panellist rated 3)*

*This would be very individual to the child and family, a double session could exhaust a child (panellist rated 2)*

*a longer session is probably required (panellist rated 3)*

*Only because my experience with interpreters is minimal. I know more time will be required but unsure of how much more time is required. (panellist rated 3)*

*this should include ax in all appropriate languages.(panellist rated 5)*

*Double time seems like too much to cover the additional time needed. Perhaps 1.5 x (panellist rated 3)*

*Ideal and recommended, but not feasible in practice (panellist rated 3)*

Statement 45: Option for the intervention to be delivered both through face to face and online sessions(within a future adaptation) (hybrid format) (EB, SG).

Percentage rated as appropriate or very appropriate: 72.2% (26/36)

Median and interquartile range: 4 (3/4)

Comments:

*May be appropriate if the child is able to access online sessions. However could also be an appropriate method of modelling a strategy/ technique to a parent and watching them carry it out (panellist rated 4)*

*Where there is a lot of time needed for discussion with parents, online might be appropriate and provide more flexibility. When working with child directly, face to face should be an option where possible. (panellist rated 4)*

*face-to-face works best for this age group.(panellist rated 2)*

*This could be a useful option, perhaps if there were parent only sessions. It is challenging carrying out therapy sessions online with this age of children (panellist rated 4)*

*great idea! (panellist rated 5)*

*There are obvious drawbacks to online sessions but if this would support some families being able to access the support where they otherwise would not then this would be a good option for those families. It may also allow input from settings as well as parents if everyone can join virtually. (panellist rated 4)*

*This would be very much dependent on the families and their access/keenness for virtual work (panellist rated 4)*

*Very family dependent (panellist rated 3)*

*I wouldn’t like this option personally but appreciate it might be useful in certain areas. (panellist rated 4)*

*Online sessions would be very difficult with children so young and it does not foster the some interaction that you can achieve face-to-face. (panellist rated 2)*

*Hanen seems to work well being delivered in this way, with one/two home visits (panellist rated 4)*

*Ideally, face to face delivery may be more appropriate (panellist rated 3)*

*Online may work for parent coaching element but not for direct work with an EY child (panellist rated 3)*

*Sounds good, but unsure how the demonstrations would go. (panellist rated 4)*

*I am concerned at how effective online delivery would be with this age group (panellist rated 3)*

*Consider access to IT; computer literacy as possible barriers- what are the options if access to online is difficult? (panellist rated 4)*

*depending on the needs of the child and the type of intervention being delivered. also consider attention/focus via online sessions as preschool age range (panellist rated 3)*

*Easier in terms of travel, can still talk through and negotiate practice or model and demonstrate/review things through telehealth. Some things will need to be f2f but it's good to be flexible. (panellist rated 5)*

*To engage the child in activities and hear what the child is saying (perception of speech sounds can be difficult on video calls), better to do face to face (panellist rated 2)*

*May increase access to service (panellist rated 4)*

*With careful consideration about which children who may benefit from online sessions, as I have experienced reduced outcomes from online sessions personally (panellist rated 4)*

*Personally I found online sessions much much less productive than face to face , especially for younger children. You are not able to observe a parent delivering therapy on a tiny mobile screen. Sound/connection quality is an issue where we are when families live out in a village. Perhaps an online, check in to see how things are going with the parent would be ok.(panellist rated 2)*

*I find this really works for a small percentage of children so should be offered as an option but not mandatory.(panellist rated 3)*

Statement 46: Option for full delivery at home, with the clinician doing home visits (within a future adaptation) (SG).

Percentage rated as appropriate or very appropriate: 66.7% (24/36)

Median and interquartile range: 4(3/5)

Comments:

*Unlikely to be able to offer this for all children (panellist rated 2)*

*Could suit some families very well but is very resource heavy in terms of therapist time so not practical in many circunstances (panellist rated 3)*

*Home visits are more time-consuming and we would be seeing fewer children per day and not meeting our service delivery targets. (panellist rated 2)*

*This would not benefit my service personally but it could be useful in some situations. I would want to prioritise linking with educational settings over this. (panellist rated 3)*

*Some families will struggle with clinic access. Also consider SLT delivering in school setting, e.g. for working parents for whom time off can be challenging (panellist rated 5)*

*I think service delivery could have an impact on this but this would be a good option where possible if this is something that would benefit families. (panellist rated 4)*

*An ideal structure but would be more time demanding and have impact on service delivery ie. see less children in one day - current home based interventions programmes in our service have caused increases to wait times and are just not feasible to run, especially within NHS - there would perhaps be more flex for this in independent practice (panellist rated 3)*

*I work in a very deprived area and I don’t think expecting families to come to clinic is realistic. (panellist rated 5)*

*This is never going to be affordable for NHS SLTs to deliver in person. So you would need a cheaper workforce to train and then you dilute the knowledge and skills. (panellist rated 1)*

*this would be lovely if Family Hub practitioners could access training to deliver at home. (panellist rated 4)*

*Could be useful for some families and the more flexibility we have on delivery with good evidence base the better (panellist rated 4)*

*Would be great to have this as in practice schools/ pre-schools often don't do the work they have been asked to do with children (panellist rated 5)*

*too close a remit for support- ideal would be across settings if possible (panellist rated 2)*

*consider feasibility within service remits. (panellist rated 3)*

*this option would be helpful although within the NHS service that i work in it would be much more likely that the child would be seen in clinic rather than at home (panellist rated 4)*

*If OK with the clinician/service, it would be good to model within the child's own environment so it's instantly applicable for the parent. (panellist rated 5)*

*in circumstances when this suits the family best and clinician is able to offer this. Could lead to better generalisation of skills at home for parents as there is not the physical and mental separation of clinic and home (panellist rated 4)*

*Flexible delivery may allow greater uptake (panellist rated 4)*

*my concern with this would be cost - home visits would take much more time than clinic-based appointments, and in that way would not be value for (public) money. Hybrid of face to face (clinic) and virtual (at home) appointments would be more feasible in my service (and we have found virtual appointments are a good way to 'see' how practice is going at home). But I can see how other services may be able to offer home visiting, so I don't have a problem with it from a clinical perspective, it's more that I don't think it's the most cost effective method of delivery (panellist rated 2)*

*Yes, with engaged and well-informed parents (panellist rated 4)*

*Lovely in theory but which trust will allow all that travel and time commitment?? I know ours wouldn't (panellist rated 2)*

**Section 3: Statements which need further clarity/specification**

***(These will re-phrased so you can re-rate round 2)***

Statement 11: Intervention techniques and activities will be explicitly linked to the target they are addressing (SG). *Rationale: By having a clear link between targets and content, parents and wider support networks will better understand the purpose of what they are doing.*

Percentage rated as appropriate or very appropriate: 97.2% (35/36)

Median and interquartile range: 5(4/5)

Comments:

***I am not sure I understand the statement (panellist rated 3)***

*it would be inappropriate to deliver interventions that are not linked to the targets (panellist rated 5)*

*Clearly defined intervention/activities to reach a goal not always detailed and I feel parents can then be 'at see' with how to implement the goals at home themselves. Explicit linking of the goal with the intervention will help this enormously (panellist rated 4)*

Statement 23: Inform about social consequences BCT: The clinician will talk to the parent about the potential social consequences (positive or negative) of carrying out the intervention techniques (EB, SG). Note: positives emphasised, negatives kept to a minimum and to be discussed sensitively. *Rationale: The parent understands why the intervention and related techniques are important. This may support with motivation.*

Percentage rated as appropriate or very appropriate: 77.8% (28/36)

Median and interquartile range: 4 (4/5)

Comments:

*I would want to discuss what are the negative consequences with another SLT colleague (panellist rated 3)*

***I'm not quite sure what this statement means? Is this linked to impact- i.e. the child will be less frustrated/ will be able to communicate with their friends more easily (panellist rated 4)***

*I think if you are going to do this, you need to be completely honest about the positives and negatives otherwise it is misleading. (panellist rated 2)*

*Need to be careful not to over-load families - everyone's life is very busy. (panellist rated 3)*

*important to have clear expectations before starting intervention; plus to problem solve around barriers.(panellist rated 4)*

*This feels like too much rationale/discussion of consequence being given for parents I work with to take in and seems implicit in the other explanations given. (panellist rated 3)*

*not something I have done in the past, perhaps because positive consequences seem obvious and may place high parental expectations on what may be very gradual change. Maybe I could try it though (panellist rated 3)*

*My experience is that parents/care-givers can become bogged down with this level of information. Whilst it is important they are engaged, I feel from experience that they re willing to accept professional direction on this (panellist rated 3)*

***not sure I understand this statement, I can see positive social consequences but negative???(panellist rated 3)***

**Section 4: Targets 2 and 3 (phonological and sound awareness)**

Our discussion with Helen Stringer regarding this was prompted by the following comments:

***Appropriate but may not be needed/priority for all. would probably do some phonological awareness if a child was struggling with other aspects of phonological discrimination*** (panellist rated 3 for target 2-phonological awareness)

***is this also considering the developmental patterns of phonological awareness (e.g., foundational word and syllable segmentation before phoneme?***  *(panellist rated 4 for target 2- phonological awareness)*

***I think this would be appropriate, but could you be more specific with what you mean by 'sound awareness'? How does this differ from phonological awareness****? (panellist rated 5 for target 3-sound awareness)*

***Too many targets will dilute the frequency and dosage; and may well confuse the family so that they embed none of the targets/strategies*** *(panellist comment after target 4)*

***This is very much dependent on whether this is an issue for the child. For some children it may not be needed*** *(panellist rated 3 for phonological awareness activities)*

***Although need to be clear if this would move up to sound level if they were already competent with syllable level phonological awareness*** *(panellist rated 4 for phonological awareness activities)*

***Too many targets at once*** *(panellist comment after content for target 4)*

**Section 5: Statements that achieved consensus**

***(You do not have to re-rate these in round 2. However- we will be taking your free text comments relating to these statements forward into the next phase of our intervention development work)***

| **Statement** | **% (n/n)** | **Median and inter-quartile range** |
| --- | --- | --- |
| 1. Target one: The intervention will include a vocabulary target which is based on both developmental norms and words the child is most likely to need within daily life (S, SG). | 97.2% (35/36) | 5 (4/5) |
| 4. Target four: The intervention will include a comprehensibility target (e.g. use of aided language boards, telling the child “show me”), based on which activities their comprehensibility is most impacting (EB, S, SG). | 88.8% (32/36) | 4(4/5) |
| 5. Set behaviour goal BCT (behaviour change technique): The parent and clinician will set goals for how the parent will carry out the intervention techniques (EB, SG). | 91.7% (33/36) | 5(4/5) |
| 6. Goal strategizing BCT: The parent and clinician will talk through barriers to implementing techniques and review strategies for overcoming these barriers (EB, SG). | 97.2% (35/36) | 5(4/5) |
| 7. Agree outcome goal BCT: The parent and clinician will agree the child’s targets (EB, SG). | 100% (36/36) | 5(5/5) |
| 8. Action planning BCT: The parent and clinician will plan when, where and how the intervention techniques will be carried out (EB, SG). | 97.2% (35/36) | 5(4/5) |
| 9. Where the child is bi/multilingual, intervention targets will align with any available norms for their home language (EB, SG) | 88.8% (32/36) | 5(4/5) |
| 10. Before setting targets, the clinician will liaise with other services (where relevant) to ascertain prior and current support received (SG) | 94.4% (34/36) | 4(4/5) |
| 12. Target one: Vocabulary will be targeted through adult exposure according to the child’s level of language development (e.g. single word modelling for minimally verbal children, match + one for early combiners) (EB, S, SG, SR). | 97.2% (35/36) | 5(4/5) |
| 15. Target four: Speech comprehensibility will be targeted through integration of strategies into everyday activities- e.g. using aided language boards at home, telling the child “show me” (EB, S, SG). | 94.4% (34/36) | 5(4/5) |
| 16. Target four: Strategies to support with speech comprehensibility in everyday life (e.g. selecting vocabulary for picture boards) will be co-produced with the child’s family (SG). | 97.2% (35/36) | 5(5/5) |
| 17. Knowledge development behaviour BCT: The Clinician will explain to the parent the rationale for intervention and what speech and language therapy is (EB, SG). | 100% (36/36) | 5(5/5) |
| 18. Provide feedback BCT (behaviour change technique): The clinician will give feedback to the parent about how they are conducting intervention techniques (EB, SG, SR). | 94.4% (34/36) | 5(4/5) |
| 19. Provide feedback on outcome of behaviour BCT: The clinician will give feedback to the parent on the impact of them conducting the intervention techniques (i.e. the change observed in the child) (EB, SG). | 100% (36/36) | 5(4/5) |
| 20. Self-monitor behaviour BCT : The parent will monitor how they are continuing with techniques/activities at home as an informal measure of progress (EB, SG). | 94.4% (34/36) | (4/5) |
| 21. Self-monitor outcome of behaviour BCT: The parent will monitor the impact of their work with their child at home as an informal measure of progress (EB, SG). | 88.9% (32/36) | 4(4/5) |
| 22. Social Support BCT : The clinician will take the time with the parent to establish who is best placed to deliver the intervention techniques, and wider family support which might facilitate intervention implementation. (EB, SG) | 91.7% (33/36) | 5(4/5) |
| 25. Demonstrate the behaviour BCT : The clinician will model techniques for the parent to see (EB, SG). | 100% (36/36) | 5(5/5) |
| 26. Reduce cue frequency BCT: The clinician will gradually withdraw prompting/cues when the parent is carrying out the technique (EB, SG). | 100% (36/36) | 5(4/5) |
| 27. Skill development behaviour BCT: The parent will practice carrying out the technique with the clinician (EB, SG). | 100% (36/36) | 5(4/5) |
| 28. Provide positive social consequence for the behaviour BCT : The clinician will provide praise when the parent is making progress with implementing the techniques (EB, SG). | 97.2% (35/36) | 5(4/5) |
| 29. Techniques will be primarily input based, with flexibility to elicit speech/language directly from the child if they demonstrate readiness for this (S, SG). | 80.6% (29/36) | 4.5(4/5) |
| 30. ‘Readiness’ for direct elicitation of speech/language will be jointly decided by the clinician and family (and child where possible) (SG). | 91.7% (33/36) | 5(4/5) |
| 31. The intervention will include a flexible option of activities/routines for parents to incorporate relevant language techniques into, with support to identify their own (SG). | 97.2% (35/36) | 5(4/5) |
| 33. The language aspects of the intervention will primarily be delivered through the supported parent using language facilitation techniques in the child’s everyday life (EB, S, SG, SR). | 100% (36/36) | 5(4/5) |
| 34. Guidance will be given regarding dosage ranges for intervention techniques (technique dosage per activity) (EB, S, SG). | 94.4% (34/36) | 5(4/5) |
| 35. Guidance will be given regarding an intervention duration (including number of sessions and spacing of sessions) (EB, S, SG, SR) | 97.2% (34/35) | 5(4/5) |
| 37. Intervention activities should be changed if the child lacks motivation or is not enjoying them (S, SG). | 97.2% (35/36) | 5(4/5) |
| 38. Add objects to the environment BCT:  Parents to bring the child’s favourite toys/books/items from home into the clinic (EB, SG). | 83.3% (30/36) | 5(4/5) |
| 39. Generalisation in learning BCT: Parents will be asked to deliver the intervention techniques from clinic at home (EB, SG). | 100% (36/36) | 5(4.25/5) |
| 40. The intervention manual will include a flexible discussion guide to support both the clinician in getting to know the family, and the family in understanding more about speech and language therapy (EB, SG). | 94.4% (34/36) | 4(4/5) |
| 41. The intervention manual will include guidance on what ‘readiness’ for direct speech work (i.e. eliciting words from the child) could look like (S, SG). | 94.4% (34/36) | 5(4/5) |
| 42. The intervention manual will include targets which can be selected across the 4 target areas, as well as guidance around progression and links to relevant non-English speech/language norm resources (SG). | 100% (36/36) | 5(4/5) |
| 43. The intervention manual will include key handouts for parents, which can be personalised (S, SG) | 100% (36/36) | 5(5/5) |
| 44. Option for the intervention to be delivered with education professionals, in educational settings (S, SG). (potential future adaptation) | 94.5% (34/36) | 5(4/5) |
| 47. Future development of a shared ‘app’ for parents and clinicians as a tool for guiding intervention delivery and monitoring progress (EB, SG). | 94.5% (34/36) | 4(4/5) |

Statements which achieved consensus but will be tweaked in response to reducing them to a single target:

| **Statement** | **% (n/n)** | **Median and inter-quartile range** |
| --- | --- | --- |
| 2. Target two: The intervention will include a phonological awareness target which is suitable for their developmental level (i.e. attention and listening capacity) (EB, S, SG). | 97.2% (35/36) | 5 (4/5) |
| 3. Target three: The intervention will include a sound awareness target, based on the child’s speech process errors (EB, SG). | 97.2% (35/36) | 5 (4/5) |
| 13. Target two: Phonological awareness will be targeted through syllable segmentation activities, or word segmentation if the child is not ready for syllable work yet (EB, S, SG). | 94.4% (34/36) | 5(4/5) |
| 14. Target three: Sound awareness relating to error processes will be targeted through hybrid use of focused auditory stimulation (also known as auditory bombardment), recasting, visual cues (e.g. cued articulation), and exposure to word contrasts (EB, S, SG, SR). | 100% (36/36) | 5(4/5) |
