# Supplementary material for: Co-design to consensus: Identifying the core elements of a novel intervention for pre-school children with co-occurring phonological speech sound disorder (SSD) and developmental language disorder (DLD) using a modified e-Delphi approach
Source: PLoS One. 2025 Jun 18;20(6):e0326072. doi: 10.1371/journal.pone.0326072 (PMC12176183; doi:10.1371/journal.pone.0326072)
Supplement: Attachment — Submitted filename: PONE-D-24-58856_comments.pdf [file pone.0326072.s009.pdf]

General comments

- Consider abbreviating Speech and Language Therapist as ‘SLT’ for consistency and brevity.
- ‘Delphi’ should always be capitalized.
- The rationale for selecting the e-Delphi methodology (lines 225–230) is well-explained, but the term ‘e-Delphi’ should be defined earlier in the manuscript, especially since it appears in the title.
- It may be more than this paper would warrant, but it could be valuable to include a figure (such as a pie chart) to illustrate the number of key elements that reached consensus in round 1 and round 2.

Line-by-line comments

36 & 156: Consider using either ‘a priori’ or ‘prior~~i~~’

71–89: DLD and SSD are clearly defined and differentiated here. While the long-term outcomes are listed, it would be helpful to include specific examples of different errors associated with DLD alone, SSD alone, and the co-occurrence of DLD and SSD.

118: ‘this is known as’

123–124: Could you provide examples of other approaches referenced here?

127: The term ‘stakeholders’–Is this referring to professionals and individuals with lived experience? Are others included in this group? Could you clarify if this is analogous to the ‘steering group’ mentioned earlier?

154: The phrase ‘To date’ – Is this referring to ongoing research?

179: ‘Recuitment’– please check spelling.

209: What were the specific probes used? Would it be possible to provide examples?

217: It may be helpful to spell out ‘BCT’ in the text, even if it's already defined in Table 1.

295–305: Approximately how long did the survey take to complete? What percentage left free text comments?

393–395: The number of key elements by group is useful, but adding percentages could provide more clarity.

407: Is ‘within the research arena’ supposed to be ‘area’?

434: ‘heterogenous’ – please check spelling.

473: ‘will need to be addressed’

Table comments

Table 1

- Page 10: Please cite Miro similarly to how you cite Qualtrics. Alternatively, add ‘whiteboard’ whenever Miro is mentioned.
- Pages 10 & 11: How were the sub-groups determined? Could you clarify who was in each group?

Table 2

- Page 20: It seems like ‘to’ or ‘with’ is missing here: ‘By agreeing to/with the child’s targets’
- Page 25: ‘Withdrawing cues will enable ~~to~~ the parent to’
- Page 27: ‘this will make the experience more positive for the parent and support with motivation.’ – should this be ‘support them with motivation,’ ‘support their motivation,’ or ‘support motivation’? (similar wording on Pages 31 & 43)

- Page 29: ‘By monitoring how they are getting on, the parent will be able to modify their input accordingly.’

Table 3

- Page 37: ‘Intervention techniques and activities will be explicitly linked to the target (or target area?) they area addressing.’ (similar phrasing on Page 42)
- Pages 38, 39, 40, & 45: Please be consistent with ‘face-to-face.’ (also Line 468)
