# Supplementary material for: Co-design to consensus: Identifying the core elements of a novel intervention for pre-school children with co-occurring phonological speech sound disorder (SSD) and developmental language disorder (DLD) using a modified e-Delphi approach
Source: PLoS One. 2025 Jun 18;20(6):e0326072. doi: 10.1371/journal.pone.0326072 (PMC12176183; doi:10.1371/journal.pone.0326072)
Supplement: Attachment — Submitted filename: R1_additional_comments.pdf [file pone.0326072.s011.pdf]

Additional questions

1. Thank you for clarifying the use of Miro whiteboards. However, is it necessary to name Miro specifically, or would a more general term such as "online whiteboard" suffice for the purposes of the Methods section?
2. Were panellists offered any form of compensation for their participation?
3. Line 293: Could you clarify the rationale behind selecting 50% as the threshold for reconsideration?

Suggestions for reducing length

Lines 175–179; 201–206:
Consider condensing each set of bullet points into a single sentence.

Table 2:
Consider simplifying Table 2 to include just two columns—Statement and Rationale. The accompanying percentage, median, and IQR data could be moved to the Supplementary Materials. While informative, this quantitative detail may be redundant in the main text since the paper already defines agreement as <75%, and the range of agreement percentages is mentioned prior to the table.

Table 3:
This is a strong and well-constructed table. However, as the primary focus of the paper is the development of the new scale, consider moving Table 3 to the Supplementary Materials. It appears to be more relevant to the Methods and may not be essential in the main body of the manuscript.
