# Supplementary material for: Co-design to consensus: Identifying the core elements of a novel intervention for pre-school children with co-occurring phonological speech sound disorder (SSD) and developmental language disorder (DLD) using a modified e-Delphi approach
Source: PLoS One. 2025 Jun 18;20(6):e0326072. doi: 10.1371/journal.pone.0326072 (PMC12176183; doi:10.1371/journal.pone.0326072)
Supplement: Attachment — Submitted filename: Response to reviewers.docx [file pone.0326072.s010.docx]

**Response to reviewers**

**Editor**

***This is an important and well-conducted study, though some concerns need to be addressed. I encourage the authors to check all comments carefully and ensure the rebuttal clearly highlights the changes made, providing justifications for both changes and aspects left unchanged. Please respond all comments and highlight in the ms.***

***Response:* Thank you for your feedback and for stating that this is an important and well conducted study. We have responded to both reviewer’s comments and have made changes accordingly. More detail is given under the ‘reviewer 1’ and ‘reviewer 2’ sections below.**

**Journal styling requirements**

1. Please ensure that your manuscript meets PLOS ONE's style requirements, including those for file naming. The PLOS ONE style templates can be found at

https://journals.plos.org/plosone/s/file?id=wjVg/PLOSOne_formatting_sample_main_body.pdf and

<https://journals.plos.org/plosone/s/file?id=ba62/PLOSOne_formatting_sample_title_authors_affiliations.pdf>

**Response:** File names checked

2. Thank you for stating in your Funding Statement. Please provide an amended statement that declares *all* the funding or sources of support (whether external or internal to your organization) received during this study, as detailed online in our guide for authors at http://journals.plos.org/plosone/s/submit-now.  Please also include the statement “There was no additional external funding received for this study.” in your updated Funding Statement. Please include your amended Funding Statement within your cover letter. We will change the online submission form on your behalf.

**Response:** Full funding statement given in the cover letter

3. We note that this data set consists of interview transcripts. Can you please confirm that all participants gave consent for interview transcript to be published?

If they DID provide consent for these transcripts to be published, please also confirm that the transcripts do not contain any potentially identifying information (or let us know if the participants consented to having their personal details published and made publicly available). We consider the following details to be identifying information:

- Names, nicknames, and initials

- Age more specific than round numbers

- GPS coordinates, physical addresses, IP addresses, email addresses

- Information in small sample sizes (e.g. 40 students from X class in X year at X university)

- Specific dates (e.g. visit dates, interview dates)

- ID numbers

Or, if the participants DID NOT provide consent for these transcripts to be published:

- Provide a de-identified version of the data or excerpts of interview responses

- Provide information regarding how these transcripts can be accessed by researchers who meet the criteria for access to confidential data, including:

a) the grounds for restriction

b) the name of the ethics committee, Institutional Review Board, or third-party organization that is imposing sharing restrictions on the data

c) a non-author, institutional point of contact that is able to field data access queries, in the interest of maintaining long-term data accessibility.

d) Any relevant data set names, URLs, DOIs, etc. that an independent researcher would need in order to request your minimal data set.

**Response:** Information added to the cover letter.

4. Please include captions for your Supporting Information files at the end of your manuscript, and update any in-text citations to match accordingly. Please see our Supporting Information guidelines for more information: http://journals.plos.org/plosone/s/supporting-information.

**Response: Captions for supporting files added to the end of the manuscript and in text citations updated to match.**

**Reviewer 1**

*The manuscript is significantly longer than a typical journal article, which may impact readability and adherence to journal guidelines. The content needs significant condensation, with a focus on the core findings and removal of unnecessary details. A more concise presentation of the key findings and arguments would enhance clarity and accessibility for readers. I recommend restructuring or condensing sections where possible while maintaining the integrity of the study. Consider focusing on the most essential details and streamlining discussions to improve the manuscript’s overall impact.*

***Response:* Thank you for volunteering your time to kindly review our article. We have removed some content to make the paper more concise. We used the two additional supplementary materials** (1.ACCORD-ACcurate COnsensus Reporting Document; 2.GRIPP- Guidance for Reporting Involvement of Patients and the Public) to inform what key information to keep in. We have also made the paper more accessible by making changes in response to reviewer 2’s comments (below).

**Reviewer 2**

*Thank you for the opportunity to review this manuscript, which presents a valuable study on determining key intervention elements for pre-school children with co-occurring SSD and DLD. The retention of participants is impressive, and the inclusion of a diverse range of parties throughout the entire process strengthens the findings. Additionally, the clear goal of translating these results into clinical guidelines is an important contribution to the field. However, while the paper provides useful insights, it would benefit from more specific examples and definitions of key terms and phrases. Although some readers may be familiar with the theories and concepts discussed, since this manuscript is being submitted to PLOS One rather than a speech-specific journal, I recommend that certain terms be more explicitly defined for a broader audience. Please see attached comments for more specific feedback.*

***Response:* Thank you for volunteering your time to review our paper and provide feedback. We are pleased that you found the inclusion of a diverse range of parties, retention of participants, and translation of the findings into clinical practice to be strengths. We have addressed your helpful comments relating to clarity within the table below.**

| **Comment/ points for enhancing clarity** | **Author response** |
| --- | --- |
| Consider abbreviating Speech and Language Therapist as ‘SLT’ for consistency and brevity. | **SLT used throughout the paper after initial acronym has been given in full in the introduction.** |
| ‘Delphi’ should always be capitalized. | **Corrected.** |
| The rationale for selecting the e-Delphi methodology (lines 225–230) is well-explained, but the term ‘e-Delphi’ should be defined earlier in the manuscript, especially since it appears in the title. | **Thank you for feeding back that our rationale for e-Delphi methodology is well-explained.**  **We have added some information to the end of our introductory paragraph:**  “Following the co-design of potential intervention elements with a diverse project steering group, consensus on these elements was sought using e-Delphi methodology. Within an e-Delphi, experts rate statements within an electronic survey, and statements meeting a minimum threshold for inclusion are taken forward” |
| It may be more than this paper would warrant, but it could be valuable to include a figure (such as a pie chart) to illustrate the number of key elements that reached consensus in round 1 and round 2. | **Thank you for this suggestion. We agree this could be a useful way of conveying the consensus rates. We are also aware of the length of the article as it is, and reviewer 1s response concerning this. You have prompted us to consider including pie charts in future presentations we give about this work-thanks again.** |
| 36 & 156:  Consider using either ‘a priori’ or ‘priori’ | ‘A priori’ a**dded in.** |
| 71–89:  DLD and SSD are clearly defined and differentiated here. While the long-term outcomes are listed, it would be helpful to include specific examples of different errors associated with DLD alone, SSD alone, and the co-occurrence of DLD and SSD. | **Thank you for feeding back that DLD and SSD are clearly defined and differentiated.**  **We have provided some additional information/references regarding the errors associated with DLD/SSD co-occurring and alone:**  “In contrast to children with DLD in isolation, children with phonological SSDs have diffiuclties with their *expressive* phonology, i.e. speech production”  “Evidence indicates that children with a co-occurring profile have a compromised linguistic system, as evidenced by their increased speech omission patterns, in contrast to features seen within a singular SSD profile” |
| **118:**  **‘this is known as’** | **Corrected.** |
| **123-124:**  Could you provide examples of other approaches referenced here? | **Added in:**  “(e.g. efficacy, implementation, partnership or target-population based approaches)” |
| **127:**  **The term ‘stakeholders’- is this referring to professionals and individuals with lived experience? Are others included in this group? Could you clarify if this is analogous to the ‘steering group’ mentioned earlier?** | Yes this is analogous to the steering group. For clarity, we have taken out the term stakeholder here (which may refer to a wider range of people), and specified:  “whereby relevant professionals and people with lived experience are involved in the co-design of the intervention” |
| 154:  The phrase ‘to date’ – is this referring to ongoing research? | **The e-Delphi is phase 3 in a 4 phase intervention development body of work. We have added detail in to help clarify:**   - “This study builds on this pre-study PPI and CE, as a part of a four-phase body of work…” - “Phases one and two included…” - “The current study, phase 3,…” |
| **179:**  **‘Recuitment’ – please check spelling** | **Corrected.** |
| **209:**  What were the specific probes used? Would it be possible to provide examples? | **We have an example of a probe within supplementary materials 3 (S3 Pre-design steering group activities) but we needed to make this clearer. We have therefore added to the text:**  “both of which are included in **S3. Pre-design steering group activities”** |
| **217:**  **It may be helpful to spell out ‘BCT’ in the text, even if it’s already defined in table 1** | **BCT spelt out again in the text.**  Individual meetings involved the identification of potential “Behaviour Change Techniques (BCTs) …. “ |
| 295-305:  Approximately how long did the survey take to complete? What percentage left free text comments? | **Unfortunately, the response Metadata on Qualtrics includes any time that the panellists had the survey open but not necessarily working on it (they had the option to complete some of the survey and come back to it later/leave it open on their browser). However, the piloting process indicated that the survey took between 45 mins to 1 hour to complete, and we have added this in:**  “The piloting process indicated that it would take between 45 minutes and an hour to complete the survey.”  “Although free text comments were optional, 86% (31/36) of panellists left a free text comment for one or more statement.” |
| **393-395:**  The number of key elements by group is useful, but adding percentages could provide more clarity. | **Percentages added:**  “Following the second round, consensus had been reached on 44 (100%) intervention elements (statements) in total; 10 core elements relating to intervention targets (22.7%), 18 core elements relating to intervention content (40.9% ), 8 core elements relating to intervention delivery (18.2% ), 4 core elements relating to the intervention manual (9.1% ), and 4 core elements regarding future adaptions (9.1% ).” |
| **407:**  **Is ‘within the research arena’ supposed to be ‘area’?** | **Changed to ‘within research’.** |
| **434:**  **‘heterogenous’-please check spelling** | **Corrected.** |
| **473:**  **‘will need to be addressed”** | **Corrected.** |
| **Table 1:**  Page 10: Please cite Miro similarly to how you cite Qualtrics. Alternatively, add ‘whiteboard’ whenever Miro is mentioned. | **In text Miro changed to “**Miro ©”  **Whiteboard stated after referring to Miro e.g “….** opportunity to add/expand on the Miro © whiteboard.” |
| **Table 1:**  Pages 10 & 11: How were the sub-groups determined? Could you clarify who was in each group? | **Added in:**  *“*Allocation to sub-groups varied between meetings. However, to ensure a spread of expertise, sub-groups were devised so that they each contained a person with lived experience (DH or SF) and a professional with equality, diversity and inclusion (EDI) expertise (M A-K or P S-T) where possible.”* |
| **Table 2:**  Page 20: It seems like ‘to’ or ‘with’ is missing here: ‘By agreeing to/with the child’s targets’ | **We have put ‘agree the child’s targets’ as putting ‘agree to/with the child’s targets’ may be interpreted as the targets have been identified by the SLT alone, implying a passive role by the parent.** |
| **Table 2:**  Page 25: ‘Withdrawing cues will enable to the parent to’ | **Corrected.** |
| **Table 2:**  Page 27: ‘this will make the experience more positive for the parent and support with motivation.’ – should this be ‘support them with motivation,’ ‘support their motivation,’ or ‘support motivation’? (similar wording on Pages 31 & 43) | **Corrected to ‘support motivation’.** |
| **Table 2:**  Page 29: ‘By monitoring how they are getting on, the parent will be able to modify their input accordingly.’ | **Corrected.** |
| **Table 3:**  Page 37: ‘Intervention techniques and activities will be explicitly linked to the target (or target area?) they area addressing.’ (similar phrasing on Page 42) | **Corrected to page 47 ‘they are’.** |
| **Table 3:**  Pages 38, 39, 40, & 45: Please be consistent with ‘face-to-face.’ (also Line 468) | **All face-to-face for consistency.** |
