# Supplementary material for: Co-design to consensus: Identifying the core elements of a novel intervention for pre-school children with co-occurring phonological speech sound disorder (SSD) and developmental language disorder (DLD) using a modified e-Delphi approach
Source: PLoS One. 2025 Jun 18;20(6):e0326072. doi: 10.1371/journal.pone.0326072 (PMC12176183; doi:10.1371/journal.pone.0326072)
Supplement: Attachment — Submitted filename: Response to reviewers PLOS ONE.docx [file pone.0326072.s012.docx]

**Reviewer 2**

Thank you for the thorough revisions. The manuscript now provides greater clarity in its framing and use of terminology, and the expanded methodological detail strengthens the transparency of the study. I particularly appreciate the added context regarding the role of stakeholders and the clarification of the e-Delphi process. At this stage, I have only a few remaining suggestions. These are included in the Reviewer Attachments. This study makes an important contribution to the literature on intervention development for children with co-occurring SSD and DLD, and I look forward to seeing it published and informing future clinical work.

| **Author’s response:** We are pleased that you found the added context and clarification of the e-Delphi process helpful. Thank you once again for your helpful feedback, we have addressed your comments below. | |
| --- | --- |
| **Point** | **Response (where located)** |
| Thank you for clarifying the use of Miro whiteboards. However, is it necessary to name Miro specifically, or would a more general term such as “online whiteboard” suffice for the purposes of the Methods section? | We have replaced with “online whiteboard” (table 1) |
| Were panellists offered any form of compensation for their participation? | They were not. However, they were given the option to have their name put in the acknowledgements section of the paper if they wished. |
| Line 293: Could you clarify the rationale behind selecting 50% as the threshold for reconsideration? | Great question. There is no “set” recommended cut off point within Delphi study guidance (Diamond et al, 2014; Trevelyan et al, 2015). We opted for 50% or higher (rather than complete exclusion) as these statements had shown some acceptability amongst the panellists and therefore had greater potential for increased appropriateness subject to re-wording. |
| Lines 175–179; 201–206:  Consider condensing each set of bullet points into a single sentence. | We have kept 175-179 (now lines 204-208) as it is as we feel that the separate numbered points clearly signpost the reader to the upcoming two separate sections (co-design + delphi) in the methodology. We have combined the bullet points into a single sentence in 201-206 (now lines 230-232) |
| Table 2:  Consider simplifying table 2 to include just two columns- statement and rationale. The accompanying percentage, median, and IQR data could be moved to the supplementary materials. While informative, this quantitative detail may be redundant in the main text since the paper already defines agreement as <75%, and the range of agreement percentages is mentioned prior to the table. | Thank you for this suggestion. Although we understand what you mean, we feel it best to leave this information on the table so that it is readily available for the reader to access and understand to what level of agreement each statement reached. This is like other tables from e-Delphi studies which have been published in the journal e.g. Curtin et al. (2024) <https://journals.plos.org/plosone/article?id=10.1371/journal.pone.0301722#sec038> |
| Table 3:  This is a strong and well-constructed table. However, as the primary focus of the paper is the development of the new scale, consider moving Table 3 to the Supplementary Materials. It appears to be more relevant to the Methods and may not be essential in the main body of the manuscript. | We understand your point and have moved this to the supplementary materials (**S7 Reworded statements)** |

**Reviewer 3**

Thank you for the opportunity to review this work! The development of intervention programs targeting both speech and language is a valuable and much-needed contribution to the field. Overall, this is a compelling project, and I appreciated the opportunity to read it. I’ve noted several areas where further clarification or editing may be helpful, and I hope these comments support your efforts to strengthen the manuscript even further.

| **Author’s response:** We are thrilled that you find this to be a compelling project and a much-needed contribution to the field. Thank you so much for taking the time to review our paper. We have addressed your helpful comments in the table below. | |
| --- | --- |
| **Point** | **Response (where located)** |
| Line 84: You introduce the term ‘phonological SSDs’. It would be helpful to clarify whether you are distinguishing between phonological and articulation disorders throughout the paper, or simply noting that SSDs can have a phonological component. As you know, many children with SSDs present with both articulatory and phonological impairments. This distinction is important, as it was unclear at times whether references to SSDs throughout the manuscript were meant to include all types or specifically phonological SSDs. For instance, at Line 237, it’s not clear whether panelists were instructed to consider only phonological SSDs when reviewing the statements. In the discussion (Line 433), you refer to children with “consistent phonological SSD”, a term not previously defined or used in the paper. The manuscript would benefit from a clear statement early on about whether your focus is exclusively on children with consistent phonological disorder. If so, consider assigning a specific term (e.g., P-SSD) to differentiate it from SSDs more broadly, and revise relevant sections in the introduction, methods, and discussion accordingly. | **Added in:** “In this paper (and study) we differentiate between phonology and articulation, as articulation refers to distorted sound productions (e.g. a lisp) which respond best to traditional articulation therapy rather than cognitive-linguistic approaches” (lines 88-91)  **Added in:** “Intervention approaches for children with P-SSD in isolation varies according to their error characteristics. For example, unlike children with consistent P-SSD errors, children with inconsistent P-SSD errors respond best to a core vocabulary approach. This variation in response to intervention has implications for intervention development, as targeting children with both consistent and inconsistent errors will likely impact intervention specificity. This study focuses on children with consistent SSD errors, who make up the largest proportion of children presenting within P-SSD” (lines 107-113)  The term P-SSD has been added throughout the paper. |
| Line 85: As currently written, the sentence suggests that speech comprehensibility is defined as “frustrations at not being understood by others around them,” which is somewhat misleading. A clearer definition of speech comprehensibility would benefit the reader. It may also be useful to briefly contrast comprehensibility with intelligibility, since intelligibility typically refers to the accuracy of speech sounds, whereas comprehensibility relates more to the listener’s ability to derive meaning from the overall message. Additionally, clarifying why comprehensibility was chosen over intelligibility in this context would strengthen this section. | **Added in:** “Speech comprehensibility refers to listener being able to derive meaning from what a child has communicated based on their speech and wider contextual cues. This contrasts to the term speech intelligibility where meaning is more closely linked to the accuracy of the child’s speech sound production^15^. The current study is concerned with comprehensibility rather than intelligibility, due to the knock on impacts on the child’s everyday life. For example, the adults around them not being able to understand their needs, leading to frustration” (lines 97-103) |
| Line 95: The phrase “referred to in day-to-day practice” is a bit vague, and the sentence reads as somewhat long. If your point is that these protocols are not commonly implemented, a clearer version might be: “However, clinical research suggests that intervention protocols are not strictly implemented in day-to-day practice. Instead, speech and language therapists…” | **Rephrased:** “However, clinical research highlights that intervention protocols are often not strictly implemented within clinical practice. Instead, speech and language therapists (SLTs) ‘mix and match’…” (lines 116-118) |
| Line 98: It would be helpful to include an example to illustrate what is meant by the “infeasibility of delivery in public services” and the competing priorities clinicians face. This is a strong point in your argument, emphasizing the importance of including SLTs in intervention development, and could be further strengthened with a more detailed description of these barriers. | **Rephrased and added to:** “Reasons for this might include infeasibility of delivery in public services, which is one consequence of implementation factors, such as the views and experiences of SLTs, not being studied within intervention development processes. Infeasibility within public services may relate to the aims (outcomes) of intervention research not necessarily reflecting what SLTs would prioritise within their practice, or the complex, clinical profiles of the children they support. The active involvement of SLTs within the development of new interventions therefore has the potential to mitigate these issues” (lines 120-127) |
| Line 156: Did you mean “a priori” here? If so, consider adjusting the phrasing for accuracy. | Rephrased to “prior” (line 191) |
| Lines 183–190: I commend you for assembling such a diverse team of contributors. Including both a parent of a child with DLD and an adult with DLD adds valuable perspectives that enrich the development process. | Thank you. We have been incredibly fortunate to have such a diverse and reflective team. |
| Table 1: Is Miro board widely known among your intended readership? This may just be a lack of knowledge on my part, but I had to look it up myself, so it may be helpful to briefly explain what it is or why it was used, or use a more common term (whiteboard) that describes the same idea. | We have taken “Miro” out and referred to it as an “online whiteboard” instead (table 1) |
| Line 340: You mention 36 panelists but describe the main employer for only 35. Could you clarify what information is available for the remaining participant? | Well spotted- thank you! This was a typo, there were 5 panellists from independent practice, not 4 (line 373). |
| Table 2, Row 1: Should this be phrased as “agree on the child’s targets” and “by agreeing on the child’s targets”? It may be a dialect difference, but I found the current wording slightly unclear. | Interesting, thank you. We do wonder if this is a dialectal difference. The clarity of this statement didn’t come up as an issue in the piloting. Although we understand your point we would rather keep the statement as it is, just because this is how we phrased it in the e-Delphi survey itself. |
| Table 2, Statement 26: There appears to be a typo in the rationale: “enable to parent” should likely be “enable the parent.” | Corrected, table 2 |
| Table 2, Statement 20: This is the first use of the abbreviation SLT, which I don’t believe has been defined earlier in the paper. Consider introducing the term when it first appears and using throughout, or changing this instance. | SLT definition added in, table 2 |
| Table 2, Statement 47: This is the only statement formatted with “Statement 47” preceding the text, whereas others list only the number in parentheses. | In response to reviewer 3’s comments, we have now changed the table so that the statement number is given before each statement (not after in parentheses). |
| Table 3, Row 1: I believe there is a typo in the reworded statement where “area” should likely be “are.” | Corrected. Please note that table 3 is now in the supplementary materials (**S7 Reworded statements)** |
| Table 1 (Behavior Change Techniques): The first mention of behavior change techniques and behavior change technique ontology appears in the Methods section. Since these are central to your intervention design, consider introducing them earlier in the Introduction to help the reader understand their significance. | **Added in:** “Additionally, behaviour change theory can be used to define how a SLT might a parent in carrying out activities at home; for example, the SLT might give the parent specific feedback on how they are performing a linguistic technique with their child in clinic^27^. Explicitly specifying BCTs is important for future study replicability, as well as for our understanding of which BCTs may be ‘active ingredients’ within the intervention (i.e. essential to the intervention being a success)**^2^**. The behaviour change technique ontology (BCTO) provides a standard terminology for BCTs, for use within the development and evaluation of complex interventions”(lines 139-146) |
| Line 421: When discussing the tension between service delivery and feasibility, could you specify which panelist responses reflected this tension? Providing explicit examples would strengthen your argument. | **Added in:** “The panellist responses reflected this tension. Round one showed that some panellists were strongly against parents not doing speech related activities at home, whereas other panellists strongly agreed that it should be the role of the SLT to provide this support in clinic. Consensus was reached….” (lines 455-458) |
| Line 423: The phrase “flexibility relating to deliverer” is unclear. Do you mean flexibility regarding how intervention is delivered or the type of intervention, or? A more explicit phrasing would improve comprehension. | **Rephrased:** “Flexibility relating to deliverer (e.g. by the SLT, parent, or both) is not routine across…” (lines 459-460) |
| Line 447: Could you clarify which specific findings you are referring to here? Although I understand what you are getting at, being more explicit would make this section more impactful and easier to follow. | **Added in:** “Therefore, the updated (psycholinguistic) target area and option of corresponding intervention techniques (e.g. syllable segmentation, speech recasting) have been kept deliberately flexible….” (lines 474-476) |
| Line 462: You note both a change in how statements were worded and a shift in how participants were asked to consider them (e.g., setting aside limitations of their own service). How can you be confident that the change in responses was due to the instruction rather than the rewording itself? It may be worth addressing this potential confound. | **Rephrased:** “There is no guarantee that the consensus of re-worded statements in round 2 was a direct result of panellists disregarding their service limitations in their responses. However, free text comments in round 1 highlighted a clear discrepancy between how SLTs might ideally carry out their practice and how this is actualised within the context of service limitations” (lines 484-488) |
| Line 494: Although your phrasing of this limitation is acceptable, I would argue that the limitation lies less in the number of panelists (as you reached your recruitment target) and more in the uneven representation of different service delivery models among them. | **Rephrased: “**One potential limitation of the e-Delphi was the representation of panellists and resulting limitations to statistical analysis. A recruitment target…” (line 541 ) |

**Reviewer 4**

Thank you for the opportunity to review this important work. The stated purpose of this study was to achieve consensus on core intervention approaches that are specific to DLD and SSD. The research question is well motivated and justified and the findings make a significant contribution to the literature surrounding intervention approaches in children who have concomitant speech and language difficulties. This work is especially important in light of the CATALISE reclassification of DLD, which highlights phonology as an important interface between DLD and SSD. As a DLD researcher myself, the relation between DLD and SSD is understudied, so this work advances an important topic of discussion. In my view, this work is well positioned at the crux of a high-impact question that is of great interest to the field. I commend the authors on tackling this important question and believe this work will be of great interest to the readership of this journal. While this is more specific to speech and language intervention, the types of intervention components are translatable to multiple clinical populations and the Delphi method is well explained. The methodological detail, while lengthy, provides a useful model for researchers following the Delphi method to conduct systematic and rigorous inquiry into clinical questions. In light of my examination of the previous reviews and author responses, my comments are minor and are offered to improve readability and add to this important question.

| **Author’s response:** We are so pleased to hear that you find our work to be of high impact and appreciate the time you have taken to review our paper and provide your very helpful comments. We have addressed them in the table below. | |
| --- | --- |
| **Point** | **Response (where located)** |
| Introduction Between lines 84-121 the authors discuss shared deficits in phonology between DLD and SSD. In my view, this section would be enhanced with a few statements on some of the research related to the mechanistic ties that might support overlapping areas of deficits. What comes to mind is recent work in sequence learning in DLD by Goffman & Gerken (2023), Benham et al., (2018), and Benham & Goffman (2022) To an extent, the procedural deficit hypothesis (Ullman & Pierpont, Ullman & Pullman) may also be a useful rationale for bridging these areas. While the work in this manuscript is not theoretical in nature, a brief discussion of some of this work may provide a useful framework for motivating the ‘theory-based intervention approaches’ you address below. Some recommended citations for consideration are:  Goffman, L., & Gerken, L. (2023). A developmental account of the role of sequential dependencies in typical and atypical language learners. Cognitive Neuropsychology, 40(5-6), 243-264.  Benham, S., Goffman, L., & Schweickert, R. (2018). An application of network science to phonological sequence learning in children with developmental language disorder. Journal of Speech, Language, and Hearing Research, 61(9), [2275-2291](tel:2275-2291).  Benham, S., & Goffman, L. (2022). A longitudinal study of the phonological organisation of novel word forms in children with developmental language disorder. International journal of speech-language pathology, 24(2), 212-223. | We found these papers very interesting, thank you for sharing them. We have added some information gleaned from the papers into our introduction and have bookmarked the papers for an article we will be developing soon about the intervention theory.  **Added in:** “Recent research using network science has highlighted phonological sequence production as a unique area of difficulty in children with DLD, providing further evidence for the link between P-SSD and DLD” (lines 95-97)  **Added in:** “Recent research suggests that this shared underlying difficulty in phonology likely occurs within the context of cross-domain difficulties in sequential learning”  (lines 137-139) |
| Methods  The authors use the term “Behaviour Change Technique” quite a bit, but I found this lacking a definition. Some examples would also be helpful. It would be helpful to define this around line 229 so readers can understand why this is a core part of the intervention process. | **Added in:** “Additionally, behaviour change theory can be used to define how a SLT might a parent in carrying out activities at home; for example, the SLT might give the parent specific feedback on how they are performing a linguistic technique with their child in clinic. Explicitly specifying BCTs is important for future study replicability, as well as for our understanding of which BCTs may be ‘active ingredients’ within the intervention (i.e. essential to the intervention being a success). The behaviour change technique ontology (BCTO) provides a standard terminology for BCTs, for use within the development and evaluation of complex interventions” (lines 139-146) |
| Methods  Table 2 would benefit from having the number of the statement on the side to the left so that it is easier to refer back to. | Statement number given before each statement. |
| Methods  On Table 3, it would be useful to add the values of consensus to the table instead of just reporting that no consensus was reached. It would be of interest to know how far off the agreement was for these statements. | Percentages/medians/IQRs added for these statements. Please note that in response to reviewer 2’s comments, this table is now in **S7 Reworded statements** |
| Methods  The rate of attrition is impressive. Did the participant who dropped out provide a reason or just did not respond to the round 2 survey? | Unfortunately, no reason was provided- they didn’t respond to the requests to complete the round 2 survey. |
| Discussion  I am curious about the distinction the authors make between articulatory-based deficits and phonological-based speech sound deficits. I find that this line can often be exceptionally vague, but this work seems to take an approach that is broad and can be applied to a number of SSDs. I think this section would benefit from a brief discussion of the application of these statements to various SSDs around line 436 when the authors mention consistent phonological SSDs. Often children with CAS, who are less consistent, also exhibit language deficits, so I am curious whether these statements would be applicable to special populations who exhibit less consistency. | We have drawn upon the work of Barbara Dodd for our definitions of phonological SSD. In response to your comments and some overlapping comments from reviewer 3, we have added some more detail into the introduction regarding this:  **Added in:** “In this paper (and study) we differentiate between phonology and articulation, as articulation refers to distorted sound productions (e.g. a lisp) which respond best to traditional articulation therapy rather than cognitive-linguistic approaches” (lines 88-91)  **Added in:** “Intervention approaches for children with P-SSD in isolation varies according to their error characteristics. For example, unlike children with consistent P-SSD errors, children with inconsistent P-SSD errors respond best to a core vocabulary approach. This variation in response to intervention has implications for intervention development, as targeting children with both consistent and inconsistent errors will likely impact intervention specificity. This study focuses on children with consistent SSD errors, who make up the largest proportion of children presenting within P-SSD” (lines 107-113)  Your comment about the potential applicability of the statements to other SSD sub-groups is very interesting and not something we had previously considered. We have added a section to the discussion relating to this:  **Added in:**  ***“Wider relevance***  This e-Delphi process, and subsequent intervention, is for pre-school children with co-occurring DLD and consistent P-SSD features. We caution against the overgeneralisation of our findings, particularly in regard to the statements relating to intervention targets and corresponding activities. However, some of the core elements identified may be relevant to interventions for other SSD sub-types and/or DLD interventions which target different outcomes. This is because supporting parents to continue with activities at home is a feature of many pre-school speech and language therapy interventions, across diagnoses. Researchers may find it helpful to draw upon our statements, particularly those relating to BCTs, when developing interventions in the future” (lines 515-524) |
